# ART1 knockdown decreases the IL-6-induced proliferation of colorectal cancer cells

**DOI:** 10.1186/s12885-024-12120-0

**Published:** 2024-03-19

**Authors:** Ting Lin, Shuxian Zhang, Yi Tang, Ming Xiao, Ming Li, Hanjuan Gong, Hailun Xie, Yalan Wang

**Affiliations:** 1 Department of Pathology, Molecular Medicine and Cancer Research Center, Basic Medicine College, Chongqing Medical University, Chongqing, 400016 P.R. China; 2 Molecular Medicine Diagnostic and Testing Center, Chongqing Medical University, Chongqing, 400016 P.R. China; 3 Department of Pathology, the First Affiliated Hospital of Chongqing Medical University, Chongqing, 400016 P.R. China

**Keywords:** ART1, Cell proliferation, Colorectal cancer, gp130, IL-6

## Abstract

Colorectal cancer (CRC) is a worldwide health concern. Chronic inflammation is a risk factor for CRC, and interleukin-6 (IL-6) plays a pivotal role in this process. Arginine-specific mono-ADP-ribosyltransferase-1 (ART1) positively regulates inflammatory cytokines. ART1 knockdown reduces the level of glycoprotein 130 (gp130), a key transducer in the IL-6 signalling pathway. However, the relationship between ART1 and IL-6 and the resulting effects on IL-6-induced proliferation in CRC cells remain unclear. The aims of this study were to investigate the effects of ART1 knockdown on IL-6-induced cell proliferation in vitro and use an in vivo murine model to observe the growth of transplanted tumours. The results showed that compared with the control, ART1-sh cancer cells induced by IL-6 exhibited reduced viability, a lower rate of colony formation, less DNA synthesis, decreased protein levels of gp130, c-Myc, cyclin D1, Bcl-xL, and a reduced p-STAT3/STAT3 ratio (*P* < 0.05). Moreover, mice transplanted with ART1-sh CT26 cells that had high levels of IL-6 displayed tumours with smaller volumes (*P* < 0.05). ART1 and gp130 were colocalized in CT26, LoVo and HCT116 cells, and their expression was positively correlated in human CRC tissues. Overall, ART1 may serve as a promising regulatory factor for IL-6 signalling and a potential therapeutic target for human CRC.

**Supplementary Information:**

The online version contains supplementary material available at 10.1186/s12885-024-12120-0.

## Introduction

Colorectal cancer (CRC) led to ∼ 900,000 fatalities worldwide in 2018 [1]. It is estimated that the global burden of CRC will increase by 60% in the next 10 years [2]. The carcinogenesis and progression of CRC are complex and involve multiple interactions amongst environmental, genetic and immune system factors [3]. One of the identified CRC risk factors is chronic inflammation [4, 5]. Additionally, certain inflammatory cytokines generated during chronic inflammation are crucial for maintaining the survival and proliferation of cancer cells [4]. Among these inflammatory cytokines, interleukin-6 (IL-6) has been proven to be a key mediator linking inflammation with the carcinogenesis and development of CRC. However, the underlying mechanisms of IL-6 in CRC are still unclear.

The level of IL-6 is significantly greater in both the tissues [6, 7] and serum samples from CRC patients [8]. IL-6 activates glycoprotein 130 (gp130), which leads to the phosphorylation of STAT3 [9–11]. Activation of the IL-6/gp130/STAT3 pathway is closely associated with the proliferation, infiltration, and metastasis of tumour cells, ultimately leading to tumour progression [6, 9, 12]. However, clinical studies thus far have not shown that anti-IL-6 monoclonal antibodies have a significant therapeutic effect on CRC. Therefore, investigating the regulatory factors associated with the IL-6-induced proliferation of CRC cells is advantageous for identifying new and effective therapeutic targets [9].

Mono-ADP-ribosyltransferase-1 (ART1) is an arginine-specific ADP-ribosylation enzyme that selectively transfers a single ADP-ribose moiety from NAD^+^ to the arginine residue of protein substrates [13–15]. ART1 has been proposed to act as an essential positive regulator of inflammatory cytokines [16–18]. The inhibition of ART1 induced by a selective arginine-dependent ART inhibitor can inhibit the release of IL-6 [17, 19]. Moreover, ART1 knockdown reduces the expression level of the gene promoter of gp130 [20]. Since gp130 is the critical node in IL-6 signalling, reducing the level of gp130 may inhibit CRC growth induced by IL-6 [12]. However, to the best of our knowledge, whether ART1 can affect the IL-6 signalling pathway in CRC has not been previously investigated.

Therefore, the aim of the present study was to investigate the effect of ART1 inhibition on CRC cells in vitro and in vivo through blockade of the IL-6/gp130 pathway. Moreover, the correlation between ART1 and gp130 in CRC cells and human CRC tissues was examined. These results may lead to finding a novel approach for the molecular targeted treatment of CRC.

## Materials and methods

### Human CRC and control tissues

A total of 54 surgically removed CRC tissue samples were included, of which 23 were CRC tissues paired with adjacent tissues. These tissues were collected in the Pathology Department of the First Affiliated Hospital of Chongqing Medical University from 2012 to 2017. The average age of these 54 patients was 59 years (range, 37 to 83 years); 29 were men, and 25 were women. All tissues were reviewed by two senior pathologists with diagnostic qualifications. The diagnostic criteria were those of the 2019 World Health Organization Classification of Tumors of the Digestive System [21]. Patients provided written consent for the use of their samples, and these samples were approved for use in this study by the Medical Research Ethics Committee of Chongqing Medical University.

### Cell lines and animals

The CT26 mouse colon cancer cell line was obtained from Professor YuQuan Wei (Sichuan University, Sichuan, China). The LoVo cell line was donated by Professor WeiXue Tang (Chongqing Medical University, Chongqing, China). The HCT116 cell line was donated by Professor Zhi Dong (Chongqing Medical University, Chongqing, China). CT26 cells and LoVo cells transfected with ART1-short hairpin (sh) RNA lentivirus (ART1-sh group) or negative control lentivirus (NC group) were successfully generated and stored in previous experiments [20, 22]. The cells were cultured in RPMI-1640 medium supplemented with 10% foetal bovine serum (FBS) and 1% penicillin/streptomycin (all from HyClone; Cytiva) at 37 °C in a 5% CO_2_ incubator.

Female BALB/c mice (*n* = 30; age, 6–8 weeks; weight, 20.7 ± 0.8 g) were obtained from the Animal Experimental Center of Chongqing Medical University (Chongqing, China) and fed in a specific pathogenfree feeding room (20–26 °C; humidity, 40-70%; 12-h/12-h light/dark cycle) with access to food and water. The animal experiments were approved by the Medical Research Ethics Committee of Chongqing Medical University.

### Immunohistochemistry

Fifty-four CRC tissues and 23 adjacent tissues were fixed in 10% formalin for 48 h at room temperature, embedded in paraffin, cut into 4 μm sections and successively immersed in xylene and 100%, 95%, 85%, or 75% ethanol for dewaxing and hydration. The samples were subsequently placed in EDTA (1:50 dilution) repair solution and heated to 95–98 °C in a microwave oven (Gree). The complete immunohistochemistry protocol was conducted according to the instructions provided by the UltraSensitive Immunohistochemistry Kit (cat. no. KIT-9710, Fuzhou Maixin Biotech Co., Ltd.). Primary antibodies (ART1, 1:500, cat. no. A10103, ABclonal Biotech Co., Ltd.; and gp130, 1:400, cat. no. 48,366, Signalway Antibody LLC) were added for incubation overnight at 4 °C. Finally, the samples were imaged under an optical microscope (BX43, Olympus). The immunohistochemistry results were scored according to Fromowitz et al. [22] as follows: positive degree score: 0, no staining; 1, light yellow; 2, brown; and 3, dark brown. Five fields were randomly observed at a magnification of 200×. In each field, the positive cells among 100 tumour cells were counted, and the average percent positive cells in five fields was taken as the percentage of positive cells in the tissue section. The range of positive scores were as follows: 0, 0–5%; 1, 6–25%; 2, 26–50%; 3, 51–75%; and 4, > 75%. The final score was determined by the positive range score plus the positive degree score as follows: <2, negative (-); 2–3 (+); 4–5 (++); and 6–7 (+++).

### Cell proliferation assay

Cells were seeded in 96-well plates (1500 cells/well) overnight and treated with different concentrations (0, 5, 10, 20, 50 and 100 ng/ml) of IL-6 (MedChemExpress) for 24, 48 or 72 h (37 °C). Then, the medium containing IL-6 was discarded, 100 µl of RPMI-1640 medium and 10 µl of Cell Counting Kit (CCK)-8 solution (Bimake.com) was added, and the cells were cultured for an additional 1 h (37 °C), after which the absorbance (A) was measured at 450 nm. The cell proliferation rate was calculated as follows: (100%) = [A of IL-6 (+) - A of blank]/[A of IL-6 (-)– A of blank] ×100%.

### Colony formation assay

Cells were seeded in 6-well plates (300 cells/well) overnight and treated with or without IL-6 (25 ng/ml) (37 °C). After 2 weeks, the cells were fixed with cold 4% paraformaldehyde (Biosharp Life Sciences) for 15 min at room temperature, followed by staining with 0.5% crystal violet solution (Beyotime Institute of Biotechnology) for 5 min. The plates were then rinsed with running water and dried before image acquisition. The minimum diameter of a colony was 0.3 mm. ImageJ software (version 1.53a; National Institutes of Health) was used to quantify the colonies.

### 5-Ethynyl-2’-deoxyuridine (EdU) assay

Cells were seeded in 96-well plates (1500 cells/well) overnight and treated with 100 µl of IL-6 (25 ng/ml) for 24 h (37 °C). EdU assays (Beyotime Institute of Biotechnology) were performed according to the manufacturer’s protocol. First, 2× EdU working solution was prepared and preheated to 37 °C. A total of 100 µl of solution was added to each well of a 96-well plate for a final concentration of 10 µM. Then, the plate was incubated in the culture chamber for an additional 2 h. After the culture medium was discarded, 100 µl of 4% paraformaldehyde (precooled to 4 °C) was added to each well, and the cells were fixed at room temperature for 15 min. After removing the fixative, 100 µl of PBS was added to each well to wash the cells 3 times for 3 min each time. After the washing solution was removed, 100 µl of PBS containing 0.3% Triton X-100 was added to each well, and the cells were incubated at room temperature for 10 min. The permeabilization solution was discarded, and 100 µl of PBS was added to each well to wash the cells 3 times for 3 min each time. Images were captured using an AMG EVOS FL microscope (Thermo Fisher Scientific, Inc.) at a magnification of 200×. The average optical density was determined from three randomly selected images using ImageJ software (version 1.53a, National Institutes of Health).

### Immunofluorescence staining

Cells were seeded in 24-well plates (3000 cells/well) overnight. For phosphorylated (p)-STAT3 staining, cells were treated with IL-6 (25 ng/ml) for 24 h (37 °C). Then, 4% paraformaldehyde was used to fix the cells for 20 min at room temperature, followed by incubation with 50 µl of normal goat serum (cat. no. AR0009, BOSTER Biological Technology Co., Ltd.) for 30 min at room temperature. The primary antibodies p-STAT3^Y705^ (1:100; cat. no. 9145, Cell Signaling Technology, Inc.), gp130 (1:100; cat. no. MAB4681-SP, Novus Biologicals, LLC) and ART1 (1:100; cat. no. ab185293, Abcam) were added for incubation at 4 °C overnight, after which the fluorescent secondary antibody (red light, DyLight 549, 1:100, cat. no. A23340, Abbkine Scientific Co., Ltd.; and green light, DyLight 488, 1:100, cat. no. A23220, Abbkine Scientific Co., Ltd.) was added for incubation at room temperature for 1 h. DAPI staining was then conducted for 5 min. Images were captured using an AMG EVOS FL microscope. The average optical density of p-STAT3 and gp130, together with the colocalization rate of gp130 and ART1, were determined from three randomly selected images using ImageJ software (version 1.53a, National Institutes of Health). The Pearson correlation and overlapping coefficient results from ≥ 30 cells from three independent experiments are shown as a bar graph (error bars, SEM).

### Western blotting

Cells or transplanted tumours from BALB/c mice were lysed in RIPA lysis buffer containing protease inhibitor cocktail (MedChemExpress) and phosphatase inhibitor cocktail (Bimake.com). A BCA protein concentration determination kit (cat. no. P0010, Beyotime Biotechnology. Co., Ltd.) was used to determine the concentration of protein. Thirty micrograms of protein from each group was loaded onto 10% SDS polyacrylamide gels and then transferred to 0.42-µm pore size polyvinylidene difluoride membranes (Cytiva). The membranes were probed with primary antibodies at 4 °C overnight and HRP-conjugated secondary antibodies at room temperature for 2 h. The primary antibodies against p-STAT3^Y705^ (1:2,000, cat. no. 9145), STAT3 (1:1000, cat. no. 9139) and c-Myc (1:1,000, cat. no. 18,583) were from Cell Signaling Technology, Inc. The primary antibodies against ART1 (1:20,000, cat. no. 66958-1-lg), cyclin D1 (1:500, cat. no. 60186-1-lg) and β-actin (1:20,000, cat. no. 66009-1-lg) were from ProteinTech Group, Inc. Bcl-xL (1:500, cat. no. a5091) was procured from Bimake.com, while gp130 (1:200, cat. no. sc376280) was obtained from Santa Cruz Biotechnology, Inc. The goat anti-mouse/rabbit HRP-conjugated secondary antibodies (1:5,000; anti-rabbit cat. no. SA00001-2; anti-mouse cat. no. SA00001-1) were obtained from ProteinTech Group, Inc. Membranes were treated using an ECL kit (cat. no. WBKLS0100, Merck Millipore) and scanned with a Storm Scanner (Amersham Pharmacia Biotech, Inc.). The original electrophoretic band in the western blot results of this study are all displayed in Supplementary Material 1.

### Reverse transcription-quantitative (RT-q) PCR

RNA was extracted from cells using TRIzol® reagent (Takara Biotechnology Co., Ltd.) according to the manufacturer’s instructions. For cDNA synthesis, 500 ng of RNA was reverse transcribed using the PrimeScript™ RT reagent Kit with gDNA Eraser (cat. no. RR047A, Takara Biotechnology Co., Ltd.). Briefly, the samples were incubated with the reverse transcription reaction solution at 37°C for 15 min, followed by treatment at 85°C for 5 s and storage at 4°C. Quantitative real-time PCR was performed using SYBR Green Master Mix (cat. no. RR420L, Takara Biotechnology Co., Ltd.) on a Bio-Rad CFX96 Real-Time PCR Detection System (Bio-Rad Laboratories, Inc.). The primers used were as follows: gp130 (mouse) forwards (F), 5’-TACGAATGGCAGCATACACAGA-3’ (F) and reverse (R), 5’-GCTAAGCAAACAGGCACGACT-3’; IL-6 (mouse) F, 5’-TAGTCCTTCCTACCCCAATTTCC-3’ and R, 5’-TTGGTCCTTAGCCACTCCTTC-3’; β-actin (mouse) F, 5’-GGCACCCAGCACAATGAA-3’ and R, 5’-GGACTCGTCATACTCCTGCTTG-3’; gp130 (human) F, 5’-CGGACAGCTTGAACAGAATGT-3’ and R, 5’-ACCATCCCACTCACACCTCA-3’; and β-actin (human) F, 5’-CCTATTCCTAGAGCTACGAGCTGCCTGAC-3’ and R, ACGCTCCAGCACTGTGTTGGCGTACAG-3’. Quantification was performed according to the methods in the literature [23].

### Mouse allograft tumour model with dextran sulfate sodium (DSS)-induced colitis

Thirty mice were randomized equally into control and colitis groups, which were given (i) DSS-free drinking water or (ii) 2% DSS (molecular weight, 36,000–50,000 Da; cat. no. M7191; MP Biomedicals, LLC) in drinking water (w/w) for 7 days followed by 1% DSS for 14 days [24]. On Day 8, 5 mice in each group were anaesthetized with 4% chloral hydrate (400 mg/kg, intraperitoneal injection) until they did not respond to tail pinching. Blood samples (ranging from 0.1 to 2 ml) were subsequently collected by amputation of the tail tip [25]. The mice were then euthanized by cervical dislocation, and colon samples were collected.

The serum level of IL-6 was detected using a Mouse Interleukin 6 ELISA Kit (cat. no. KLC004.96, Kangling Biotechnology Co., Ltd.). according to the manufacturer’s protocol. First, 50 µl of standard solution (with a concentration of 120, 60, 30, 15, 7.5, or 3.75 pg/ml) was added to the standard wells of the microplate. Forty microlitres of sample diluent was added to the sample wells, followed by the addition of 10 µl of mouse serum and incubation at 37 °C for 30 min. The samples were washed 5 times, and each sample was patted dry with filter paper until no trace of water was visible. Then, 50 µl of enzyme-labelled reagent was added to each well. Next, 50 µl of each chromogenic reagents A and B was added to each well, and the plates were placed in a 37 °C constant temperature incubator for incubation in the dark for 10 min. Then, 50 µl of stop solution was added to each well to terminate the chromogenic reaction. The OD was subsequently measured at 450 nm using a microplate reader. A standard curve was prepared from the concentrations and OD values of the standard solutions from which the concentration of IL-6 in each sample was calculated.

H&E staining of the colon was conducted as follows: The samples were stained with haematoxylin for 30 s at room temperature, differentiated with 75% hydrochloric acid in alcohol and stained with eosin for 20 s at room temperature. Additionally, on Day 8, the remaining 10 mice in the colitis group were divided equally into cancer and control groups. NC- and ART1-sh-transfected CT26 cells were harvested and adjusted to a concentration of 1 × 10^7^ cells/ml. The CT26 cell suspension (200 µl, containing 2 × 10^6^ cells) [26] was subcutaneously injected into the right flank region of each anaesthetized cancer group mouse, while the control group received the same dose of phosphate-buffered saline (PBS). After 2 weeks, the mice were euthanized by cervical dislocation, and the transplanted tumours were collected. The tumour volume (V) was computed from the diameters measured with a digital calliper using the Formula *V = ab*^2^/2, where *a* is the maximum diameter and *b* is the minimum diameter.

### Statistical analysis

The measured data are presented as the mean ± SEM from at least three independent experiments. All statistical analyses were performed using SPSS (version 20.0, IBM Corp.) or GraphPad Prism 5 (GraphPad Software, Inc.). Unpaired t tests, one-way ANOVA (post hoc test: Tukey), Wilcoxon rank sum tests, Mantel–Haenszel tests or Spearman correlation analyses were used to determine the level of significance. *P* < 0.05 was considered to indicate a statistically significant difference.

## Results

### IL-6 induces cell proliferation via the gp130/STAT3 signalling axis

**Fig. 1:**
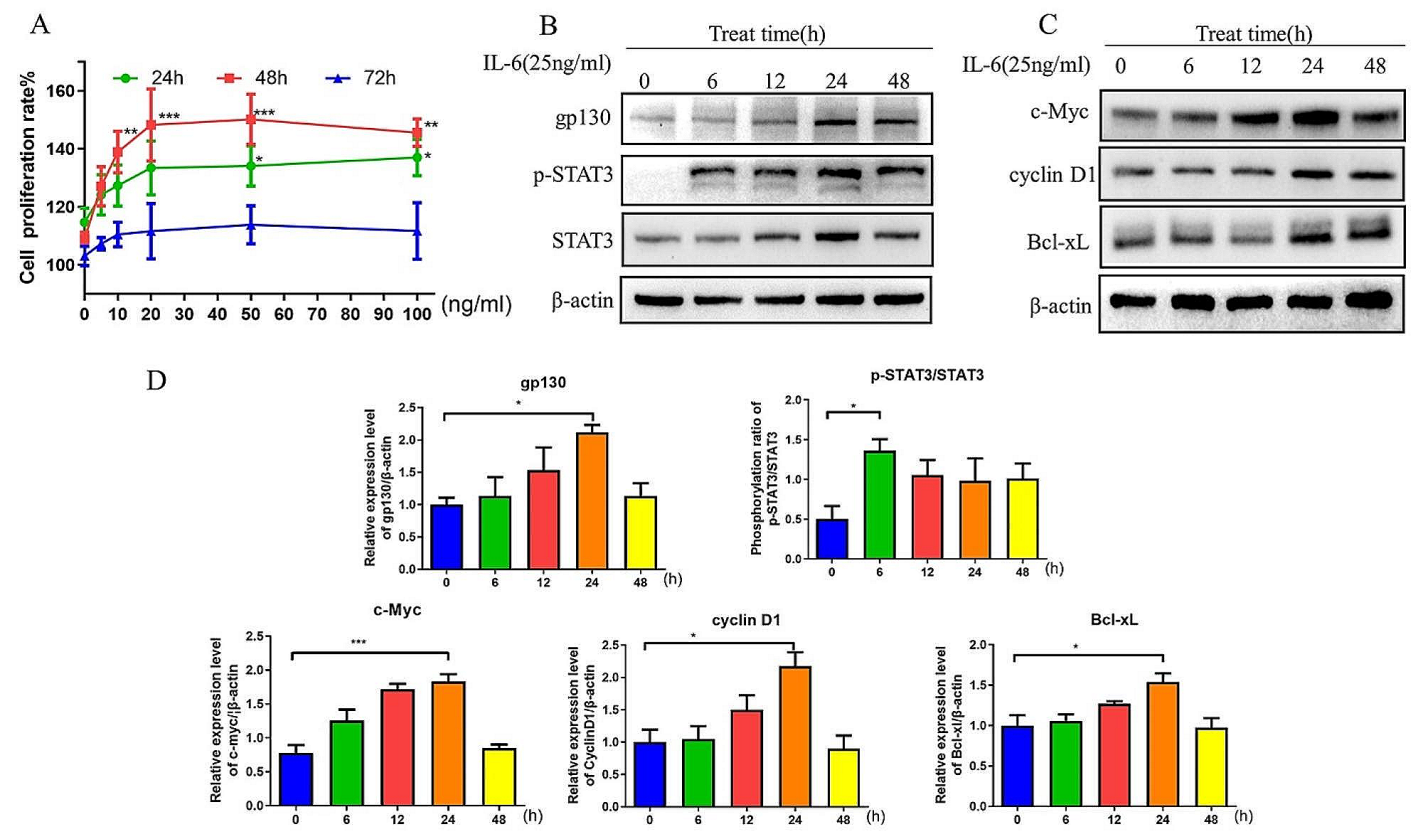
IL-6 induced the proliferation of CT26 cells. (**A**) Proliferation rates of CT26 WT cells after incubation with different concentrations of IL-6 (0, 5, 10, 20, 50 and 100 ng/ml) for different durations (24, 48 and 72 h). (**B-D**) Expression levels of gp130, c-Myc, cyclin D1, and Bcl-xL and the p-STAT3/STAT3 ratio in CT26 WT cells after induction with IL-6 (25 ng/ml) for different durations. ^*^P < 0.05, ^**^P < 0.01, ^***^P < 0.001. WT, wild-type; p-, phosphorylated; gp130, glycoprotein 130

The promotion of CT26 colon cancer cell proliferation induced by IL-6 was examined first. Within 48 h of IL-6 treatment, the cell proliferation rate increased in a dose- and time-dependent manner (*P* < 0.01) (Fig. 1A). Western blot analysis of the expression of proliferation-related proteins in the IL-6 signalling pathway was performed. The expression levels of gp130 and the target proteins c-Myc, cyclin D1 and Bcl-xL and the STAT3 phosphorylation ratio (p-STAT3/STAT3) increased with prolonged IL-6 stimulation and peaked at 24 h (*P* < 0.05) (Fig. 1B-D). Moreover, the gp130 level and the p-STAT3/STAT3 ratio remained relatively high at 48 h (*P* < 0.05) (Fig. 1C&D).

### ART1 knockdown decreases the IL-6-induced cell proliferation rate, DNA synthesis, colony formation and the protein expression levels of c-Myc, cyclin D1 and Bcl-xL

**Fig. 2:**
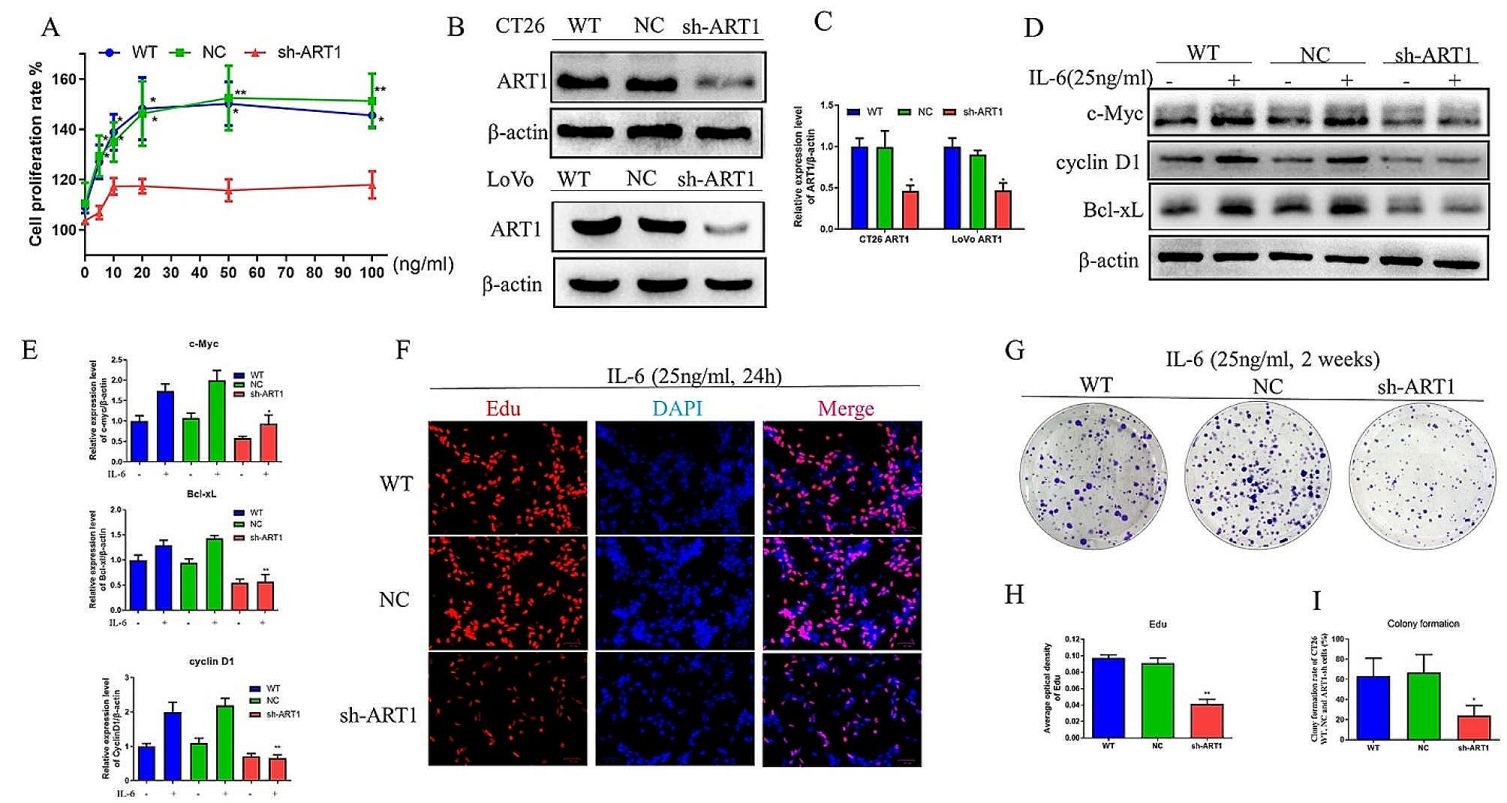
ART1 knockdown decreased IL-6-induced cell proliferation. (**A**) Proliferation rates of WT, NC and ART1-sh CT26 cells after incubation with different concentrations of IL-6 (0, 5, 10, 20, 50 and 100 ng/ml) for 48 h. (**B** and **C**) Protein expression levels of ART1 in CT26 and LoVo cells transfected with ART1-sh. (**D** and **E**) Protein expression levels of c-Myc, cyclin D1 and Bcl-xL in WT, NC and ART1-sh CT26 cells after induction with IL-6 (25 ng/ml) for 24 h. (**F** and **H**) DNA synthesis in WT, NC and ART1-sh CT26 cells after incubation with IL-6 (25 ng/ml) for 24 h. Red fluorescence represents DNA synthesis, and blue fluorescence represents the nuclei. The scale bar is 50 μm. (**G** and **I**) Colony formation rate of WT, NC, and ART1-sh CT26 cells after incubation with IL-6 (25 ng/ml) for 2 weeks. ^*^P < 0.05, ^**^P < 0.01. WT, wild-type; sh, short hairpin RNA; NC, negative control; ART1, mono-ADP-ribosyltransferase-1

To determine whether ART1 affects IL-6-induced cell proliferation, the proliferation rates of wild-type and NC- and ART1-sh-transfected CT26 cells after incubation with different concentrations of IL-6 (0, 5, 10, 20, 50 and 100 ng/ml) for 48 h was measured. The results demonstrated that, compared with that in the control group, the IL-6-induced proliferation of ART1-knockdown CT26 cells was lower at every concentration (Fig. 2A-C). Consistent with these results, the effect of IL-6 on DNA synthesis ability and colony formation rate were less in the ART1-knockdown CT26 cells compared with those in the control cells (Fig. 2F-I). To further determine the effect of ART1 knockdown on the target genes of p-STAT3, the protein expression levels of c-Myc, cyclin D1 and Bcl-xL were determined via western blotting. The IL-6-induced expression levels of cyclin D1 and Bcl-xl in the ART1 knockdown group were lower than those in the control groups (Fig. 2D&E).

### Knocking down ART1 inhibits IL-6-induced cell proliferation by downregulating gp130 and p-STAT3 expression

**Fig. 3:**
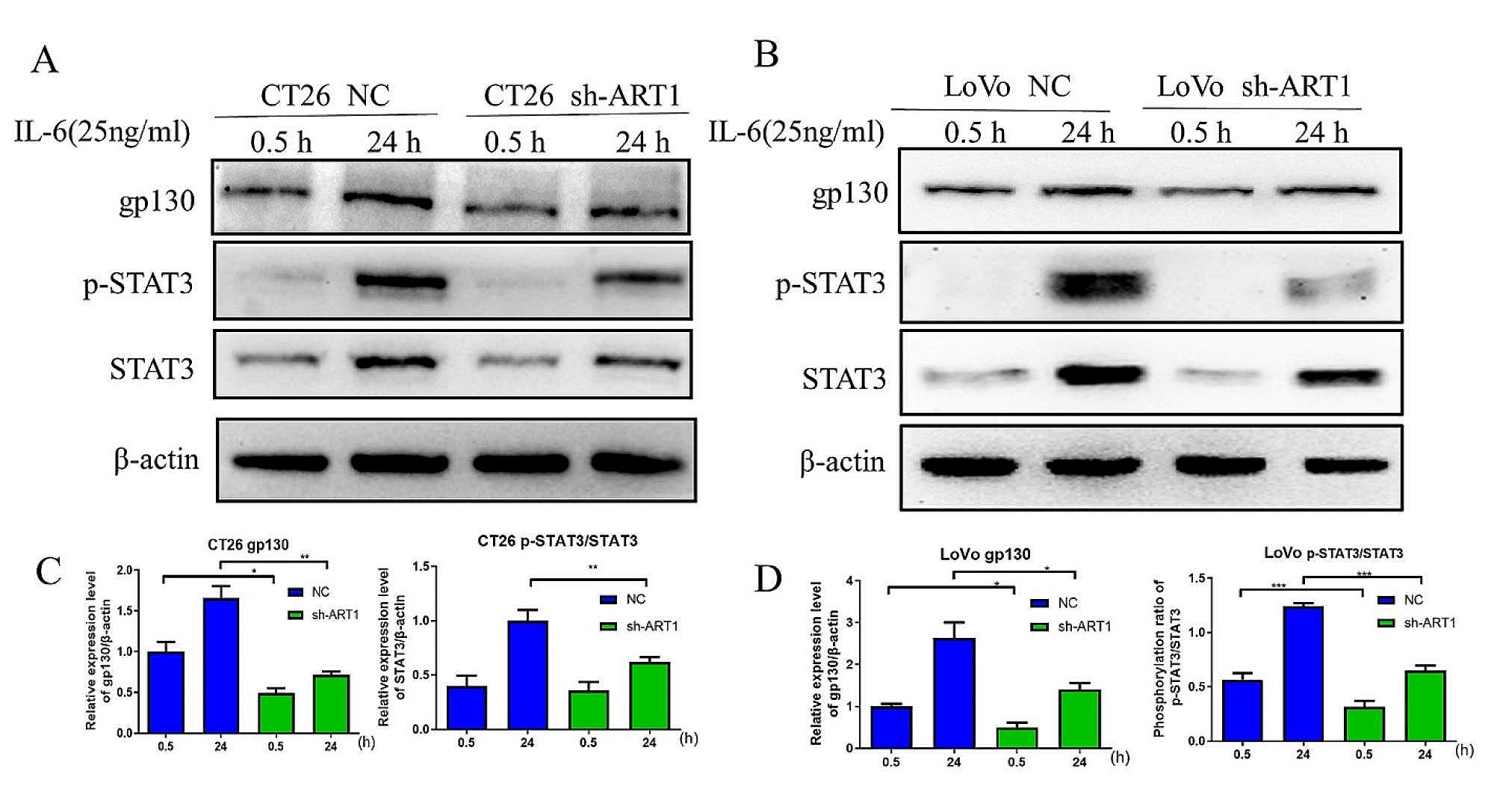
ART1 knockdown inhibited IL-6-induced gp130 and p-STAT3 expression. (**A** and **C**) Protein expression level of gp130 and ratio of p-STAT3/STAT3 in NC and ART1-sh CT26 cells after induction with IL-6 (25 ng/ml) for 0.5 or 24 h. (**B** and **D**) Protein expression level of gp130 and the p-STAT3/STAT3 ratio in NC and ART1-sh LoVo cells after induction with IL-6 (20 ng/ml) for 0.5 or 24 h. ^*^P < 0.05, ^**^P < 0.01, ^***^P < 0.001. WT, wild-type; p-, phosphorylated; gp130, glycoprotein 130; sh, short hairpin RNA; NC, negative control; ART1, mono-ADP-ribosyltransferase-1.

**Fig. 4:**
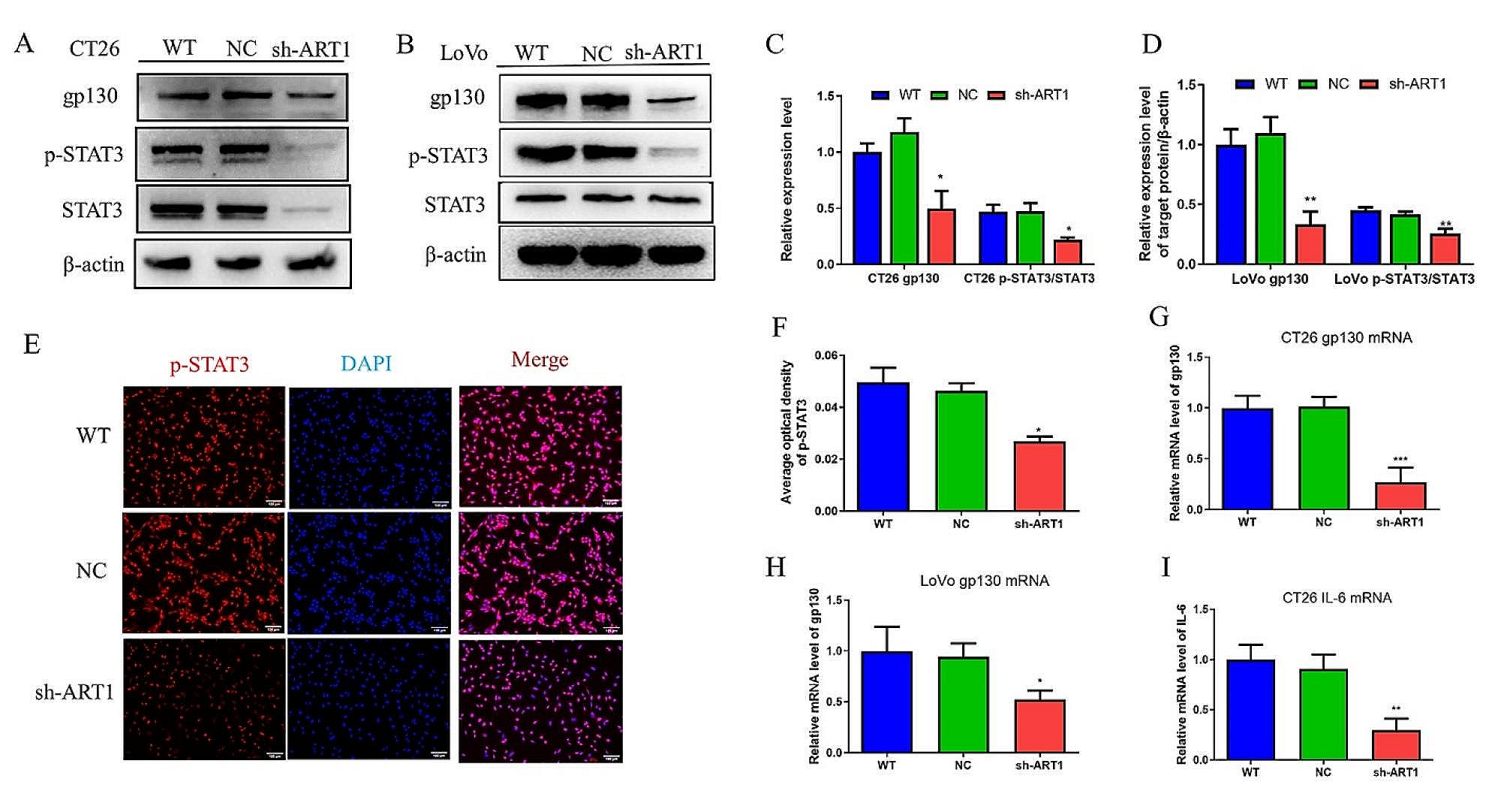
Knocking down ART1 influenced the IL-6 signalling pathway at the molecular level. (**A-D**) Protein expression level of gp130 and ratio of p-STAT3/STAT3 in WT, NC and ART1-sh CT26 and LoVo cells. (**E** and **F**) Immunofluorescence staining of p-STAT3 in WT, NC and ART1-sh CT26 cells. Red fluorescence represents p-STAT3 in both the nucleus and cytoplasm, and blue fluorescence represents the nuclei. The scale bar is 100 μm. (**G** and **H**) mRNA expression level of gp130 in WT, NC and ART1-sh CT26 and LoVo cells. (**I**) mRNA expression level of IL-6 in WT, NC and ART1-sh CT26 cells. ^*^P < 0.05, ^**^P < 0.01, ^***^P < 0.001. WT, wild-type; p-, phosphorylated; gp130, glycoprotein 130; sh, short hairpin RNA; NC, negative control; ART1, mono-ADP-ribosyltransferase-1.

To determine whether the inhibition of IL-6-induced proliferation caused by ART1 knockdown occurs via the downregulation of gp130 and p-STAT3. CT26 and LoVo colon cancer cells were treated with 25 ng/ml IL-6 for different durations (0.5 and 24 h). The protein expression levels of gp130 and p-STAT3 were evaluated after incubation with IL-6, and the fluorescence of IL-6-induced p-STAT3 was also observed. The protein expression level of gp130 and the p-STAT3/STAT3 ratio after IL-6 induction were lower in the ART1 knockdown group than in the control group (*P* < 0.05) (Fig. 3A-D). Moreover, the immunofluorescence results revealed that the levels of p-STAT3 in the cytoplasm and nucleus were lower in the ART1-knockdown CT26 cells than in the control cells (*P* < 0.05) (Fig. 4E & F).

### ART1 knockdown attenuates IL-6 signalling via gp130

**Fig. 5:**
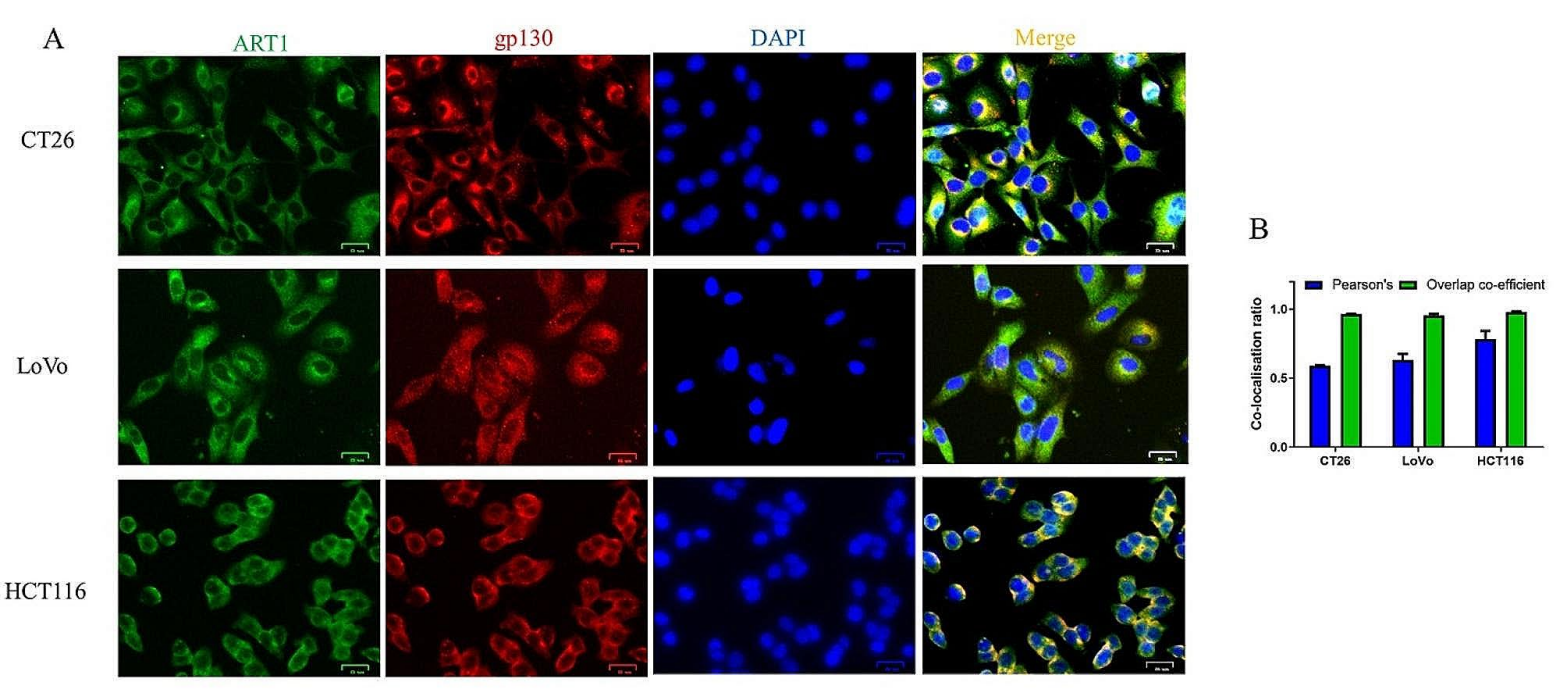
Double immunofluorescence staining of ART1 and gp130. (**A** and **B**) Double immunofluorescence staining of ART1 and gp130 in WT CT26, LoVo and HCT116 cells. Green fluorescence represents ART1, red fluorescence represents gp130, and blue fluorescence represents the nuclei. The scale bar is 25 μm. The Pearson correlation coefficients were 0.588, 0.632 and 0.782, respectively. WT, wild-type; gp130, glycoprotein 130; ART1, mono-ADP-ribosyltransferase-1.

**Fig. 6:**
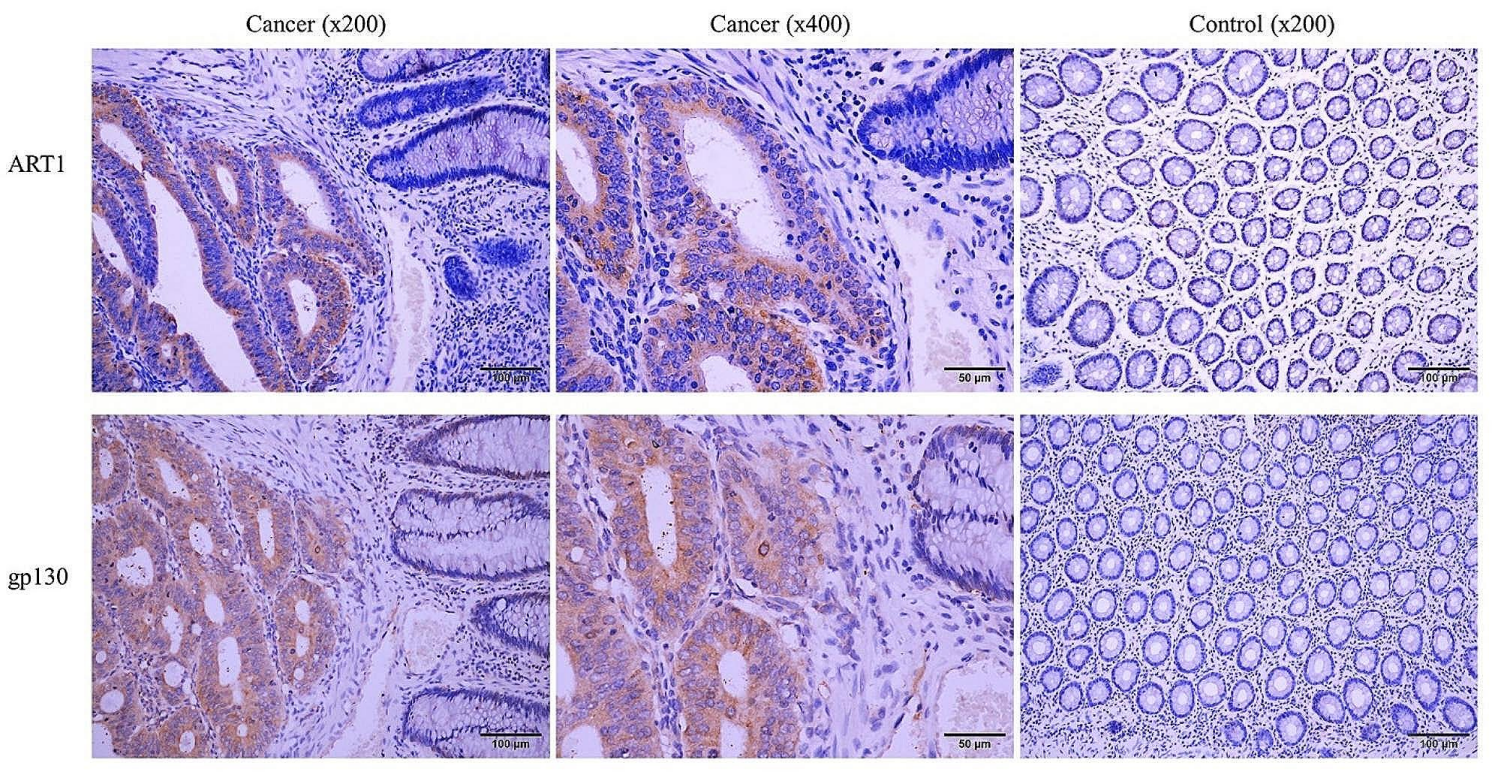
Expression levels of ART1 and gp130 in colorectal cancer tumour and control tissues. The scale bars are 50 and 100 μm. gp130, glycoprotein 130; ART1, mono-ADP-ribosyltransferase-1.

Previous research revealed that ART1-sh-transfected CT26 cells had lower p-Erk2 expression than control cells [20]. Additionally, p-Erk2 has been shown to control the expression and function of gp130 in several malignant tumour cells lines [27]. Therefore, the aim of the current study was to determine whether ART1 influences gp130. First, both the mRNA and protein levels of gp130 and the mRNA level of IL-6 were reduced in the ART1-knockdown group (*P* < 0.05) (Fig. 4A-D & G-I). Since gp130 needs to complete N-terminal glycosylation in the endoplasmic reticulum (ER) [28] and because ART1 can modify the ER [14], the colocalization of these proteins were next investigated. Immunofluorescence analyses confirmed the colocalization of ART1 and gp130 in the examined colon cancer cell lines (Fig. 5A & B). The relationship between ART1 and gp130 in human CRC tissues was subsequently determined. The immunohistochemistry results indicated that the percentage of tumour tissue positive for these two factors was greater than that in adjacent tissue (*P* < 0.01) (Fig. 6; Tables 1 and 2). The degree of positivity for ART1 and gp130 was related to the occurrence of lymph node metastasis and the pathological grade (*P* < 0.05) (Tables 3 and 4). The expression levels of ART1 and gp130 in tumour tissue were also positively correlated (*P* < 0.01) (Table 5).

**Table 1:** Expression of ART1 in CRC tumour and control tissues

Tissue	ART1	ART1	χ^2^	P
	(-)	(+)		
Cancer	5	49	40.45	*P* < 0.001
Normal	19	4		

ART1: arginine-specific mono-ADP-ribosyltransferase-1; CRC: colorectal cancer

**Table 2:** Expression of gp130 in CRC tumour and control tissues

Tissue	gp130	gp130	χ^2^	P
	(-)	(+)		
Cancer	9	45	30.31	*P* < 0.001
Normal	19	4		

gp130: glycoprotein 130; CRC: colorectal cancer

**Table 3:** Correlations between the degree of ART1 expression and clinicopathological factors in 54 patients with CRC

Clinical pathological parameter	n	ART1 positive degree				P
		-	+	++	+++	
**Total**	54	5	9	21	19	
**Age (years)**						0.244
< 59	25	3	5	10	7	
≥ 59	29	2	4	11	12	
**Gender**						0.804
Male	29	3	5	11	10	
Female	25	2	4	10	9	
**Tumour location**						0.821
Right colon	17	2	3	6	6	
Left colon	37	3	6	15	13	
**Pathology grade**						0.003^*^
Low	38	5	7	18	8	
High	16	0	2	3	11	
**Lymph node metastasis**						0.034^*^
Yes	24	0	3	10	11	
None	30	5	6	11	8	
**Metastasis**						0.121
Yes	13	0	1	6	6	
None	41	5	8	15	13	

ART1: arginine-specific mono-ADP-ribosyltransferase-1; CRC: colorectal cancer. *: *P* < 0.05

**Table 4:** Correlations between the degree of gp130 expression and clinicopathological factors in 54 patients with CRC

Clinical pathological parameter	n	ART1 positive degree				P
		-	+	++	+++	
**Total**	54	9	5	20	20	
**Age (years)**						0.680
< 59	25	5	2	7	11	
≥ 59	29	4	3	13	9	
**Gender**						0.251
Male	29	4	1	12	12	
Female	25	5	4	8	8	
**Tumour location**						0.110
Right colon	17	5	1	7	4	
Left colon	37	4	4	13	16	
**Pathology grade**						0.003^*^
Low	38	8	5	16	9	
High	16	1	0	4	11	
**Lymph node metastasis**						0.018^*^
Yes	24	2	0	10	12	
None	30	7	5	10	8	
**Metastasis**						0.143
Yes	13	0	1	6	6	
None	41	9	4	14	14	

gp130: glycoprotein 130; CRC: colorectal cancer. *: *P* < 0.05

**Table 5:** Correlation between the expression levels of ART1 and gp130

		ART1				Total	r	P
		-	+	++	+++			
gp130	-	5	3	1	0	9		
	+	0	1	4	0	5		
	++	0	3	11	6	20	0.632	< 0.001
	+++	0	2	5	13	20		
Total		5	9	21	19	54		

ART1: arginine-specific mono-ADP-ribosyltransferase-1; gp130: glycoprotein 130

### ART1 knockdown inhibits tumour growth in a colitis mouse model with elevated IL-6

**Fig. 7:**
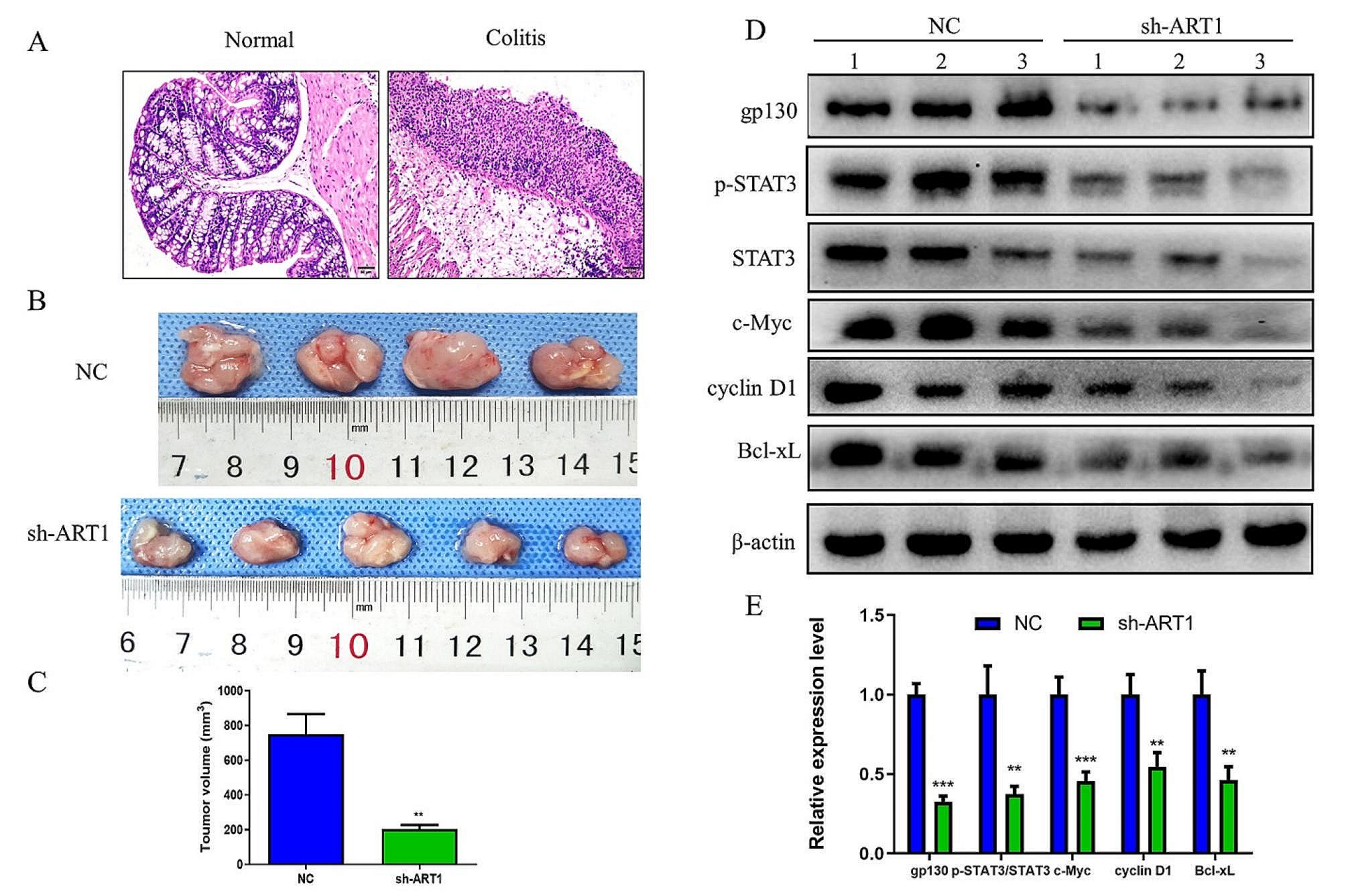
ART1 knockdown inhibits tumour growth in a colitis mouse model. (**A**) H&E staining of normal and colitis mouse colon tissues. The scale bar represents 50 μm. (**B** and **C**) Volume of transplanted tumours formed in colitis model mice by NC and ART1-sh CT26 cells. (D and E) Protein expression levels of gp130, c-Myc, cyclin D1, and Bcl-xL and the ratio of p-STAT3/STAT3 in the transplanted tumours formed by NC and ART1-sh CT26 cells in colitis model mice. ^**^P < 0.01, ^***^P < 0.001. p-, phosphorylated; gp130, glycoprotein 130; sh, short hairpin RNA; NC, negative control; ART1, mono-ADP-ribosyltransferase-1.

A previous study showed that IL-6 levels were elevated in a DSS-induced colitis model mice [29]. To determine whether ART1 knockdown can inhibit the IL-6 signalling pathway and thus inhibit IL-6-induced tumour growth in vivo, allografts were established in colitis model mice (Fig. 7A). The IL-6 levels in the colitis mouse model serum were significantly increased after 7 days of DSS treatment (Table 6). Furthermore, ART1 knockdown significantly suppressed tumour growth, as evidenced by a decreased tumour volume. The maximum tumour size in the NC group was 1,093.7 mm^3^, while the maximum tumour size in the ART1-sh group was 244.6 mm^3^ (*P* < 0.01) (Fig. 7B & C). The expression levels of gp130 and the downstream targets c-Myc, cyclin D1 and Bcl-xL and the p-STAT3/STAT3 ratio were lower in the ART1-sh-transplanted tumours than in the NC tumours (*P* < 0.01) (Fig. 7D & E).

**Table 6:** Serum levels of IL-6 in normal and colitis mice (‾x ± SD)

Group	IL-6 (pg/ml)
Normal	13.27 ± 1.50
Colitis	102.49 ± 7.51***

IL-6: Interleukin 6. ***: *P* < 0.001

## Discussion

As early as 1998, IL-6 levels were found to be greater in CRC tumour tissues than in normal control tissues [7]. Subsequently, it was reported that serum IL-6 levels are also significantly increased in patients with CRC and positively correlated with tumour size [8, 30]. In vitro experimental data revealed that IL-6 can promote the proliferation of CRC cells extracted in situ [31]. The present study also demonstrated that IL-6 promoted the proliferation of CT26 cells (Fig. 1A), which is consistent with previous reports [31, 32]. Although clinical and experimental data strongly suggest the contribution of IL-6 signalling to CRC growth [9, 32, 33], anti-IL-6 monotherapy appears to have no clinical effect on CRC [34]. Currently, the reasons for the ineffectiveness of anti-IL-6 monotherapy for CRC treatment remain unclear [33]. Angevin et al. [34] suggested that the ineffectiveness of anti-IL-6 monotherapy in CRC may be associated with autocrine IL-6 in tumour cells. As previously reported, IL-6 binds to receptors on the surface of tumour cells and activates gp130 to phosphorylate STAT3. After p-STAT3 enters the nucleus, it can activate the transcription of IL-6 and subsequently form a IL-6/gp130/STAT3 positive feedback loop [35]. Thus, identifying other regulators controlling the IL-6/gp130/STAT3 feedback loop in CRC may lead to the development of novel therapeutic options. The present study revealed that ART1 was crucial for maintaining the IL-6-induced activation of STAT3 (Figs. 3A-D and 4E, amp and F); therefore, targeting ART1 may be an effective way to disrupt the IL-6/gp130/STAT3 positive feedback loop.

gp130 is a crucial node in the IL-6 signalling pathway. It has been reported that inhibiting gp130 expression can enhance the antitumour effect of 5-fluorouracil in CRC cells, including increasing apoptosis and decreasing invasion and metastasis [36]. Moreover, gp130 degradation induced by epirubicin exerts antitumour effects on CRC HCT116 cells [37]. However, gp130 is ubiquitously expressed in all tissues and is required for myocardial remodelling [38], neuronal protection and to fight infection [39]; thus, directly targeting this protein is challenging. In the present study, a new therapeutic target, ART1, was suggested for regulating the expression level of gp130 in CRC cells. The current results indicated that IL-6 can induce the expression of gp130 (Fig. 1B & D) in CT26 cells and that ART1 knockdown can effectively inhibit this type of induction in vitro and in vivo (Figs. 3A-D and 7D, amp and E). The present study revealed a positive correlation between the expression of ART1 and gp130 in CRC tissues and that these factors colocalize in several CRC cell lines (Figs. 5 and 6; Table 5). The degree of positivity for ART1 and gp130 was related to the extent of lymph node metastasis and the pathological grade of the CRC tissues (Fig. 6; Tables 1 and 2). The results also revealed that ART1 knockdown reduced both the mRNA and protein expression levels of gp130. Therefore, ART1 may regulate gp130 at both the transcriptional and posttranslational levels. On the one hand, ART1 knockdown reduces the expression level of p-Erk2 [20], which is a promoter of gp130 [27], decreasing gp130 transcription. On the other hand, ART1 plays a modifying role both in the ER [14] and in the cell membrane [40]. Furthermore, gp130 first undergoes N-linked glycosylation in the ER, after which it can be transported to the cell membrane [28]. As a result, ART1 may interact with gp130 at the ER or cell membrane, which in turn affects the posttranslational modification of gp130. However, the mechanisms underlying these two processes require further in-depth study.

Activation of the STAT3 pathway is a key downstream signalling event in IL-6/gp130 signalling. When IL-6 binds to its receptor, gp130 dimerizes on the cell membrane where it subsequently phosphorylates STAT3 to form the dimer p-STAT3, which can function as a transcription factor [41]. It has been reported that p-STAT3 can be detected in 72% of CRC tissues, which is significantly greater than its level of detection in control tissues [42]. In glioma stem cells, inhibiting gp130 can attenuate IL-6-induced p-STAT3 expression, thereby inhibiting tumour growth [43]. In CRC cells, as the cell membrane signal transduction molecule gp130 was inhibited, the expression levels of STAT3 and p-STAT3 decreased, and cell proliferation was inhibited [44]. p-STAT3 promotes tumour growth mainly by promoting the transcription of cell proliferation and survival genes, such as *c-Myc, cyclin D1* and *Bcl-xL* [33]. The present study demonstrated that ART1 knockdown significantly inhibited IL-6-induced p-STAT3 and its target proteins, c-Myc, cyclin D1 and Bcl-xL (Fig. 2D, amp and E, 3 A-D, 7D & E), the proliferation of CRC cells (Fig. 2A-C, F-I) and the growth of transplanted tumours in a mouse model (Fig. 7B & C). Interestingly, ART1 knockdown reduced the transcription of IL-6 (Fig. 4I), which was consistent with the findings of a previous study [17]. Taken together, these data suggested that ART1 knockdown may abolish the positive feedback loop between IL-6, gp130 and p-STAT3 in CRC cells. This disruption is highly important for inhibiting tumour growth. Notably, the current study revealed that ART1 knockdown significantly reduced p-STAT3 expression in transplanted tumours in a DSS-induced colitis mouse model. DSS-induced colitis model animals have been reported to have elevated levels of several proinflammatory cytokines, including IL-6 and IL-11 [29, 45], both of which are associated with CRC development via p-STAT3. Therefore, ART1 knockdown can attenuate not only IL-6-induced p-STAT3 but also IL-11 and other STAT3-activating cytokines-induced p-STAT3, thereby inhibiting CRC growth.

ART1 is a type of enzyme that belongs to a family that is involved in posttranslational modifications and can regulate protein functions and interactions. Although research regarding the effects of ART1 on cancer is limited, current experimental evidence suggests that ART1 plays an important role in hepatocellular carcinoma [46], glioma [47] and CRC [48, 49]. Consistent with these findings, previous studies demonstrated that ART1 can promote the proliferation, angiogenesis, invasion and metastasis of CRC cells [48–50]. The present study also indicated that ART1 can promote IL-6-induced cell proliferation (Fig. 1A), while knocking down ART1 had the opposite effect (Fig. 2A-C). Thus, ART1 may serve as a new regulator of the IL-6 signalling pathway in CRC. As ART1 is a cell surface protein, it may be relatively easy to target. Systematic inhibition of ART1 may not have severe side effects; for example, ART1-knockout mice are viable and develop normally [51]. The most likely drug to use to inhibit ART1 is meta-iodobenzylguanidine (MIBG), a competitive inhibitor of ART1 that decreases the expression of ART1 [17]. Moreover, multiple rounds of MIBG treatment were associated with prolonged survival in patients with pulmonary and gastroenteropancreatic neuroendocrine tumours [52]. Interestingly, MIBG can inhibit the production of IL-6 by inhibiting ART1 [17]. However, whether MIBG can be used to treat CRC and regulate IL-6 signalling via ART1 requires in-depth research. Taken together, these findings suggested that ART1 may be a promising therapeutic option for CRC, although further validation experiments are needed.

In conclusion, the present study demonstrated that IL-6 can promote the proliferation of CT26 cells by increasing the gp130 protein level, the p-STAT3/STAT3 ratio and the expression of c-Myc, cyclin D1 and Bcl-xL. Knocking down ART1 can inhibit IL-6-induced proliferation by reducing the levels of these corresponding factors. Disrupting ART1 to interrupt the IL-6/STAT3 signalling axis may have therapeutic potential for improving CRC treatment efficacy, and the ART1-specific inhibitor MIBG has the potential to be an effective drug for this purpose. In our future work, we intend to investigate how ART1 can affect the posttranslational modification of gp130 and whether MIBG can affect the IL-6 signalling pathway by inhibiting ART1.

### Limitations

This study is a preliminary investigation into the effects of ART1 on the IL-6-induced proliferation of colorectal cancer cells and its potential underlying mechanisms. Two common types of colon cancer cell lines were selected as the research subjects, along with colorectal cancer tissue specimens from a hospital. In future studies, it is advisable to conduct a multicentre study to further investigate this topic comprehensively. In the animal experiment, we chose to establish a colitis model. Although the IL-6 concentration increased after model construction, this change was accompanied by an increase in other inflammatory factors [29, 45]. However, there is currently no better mouse model to use for this purpose, and hopefully, in future experiments, we can obtain a mouse model in which only IL-6 is elevated.

### Electronic supplementary material

Below is the link to the electronic supplementary material.

Supplementary Material 1

## Data Availability

No datasets were generated or analysed during the current study.
