# Supplementary material for: ART1 knockdown decreases the IL-6-induced proliferation of colorectal cancer cells
Source: BMC Cancer. 2024 Mar 19;24:354. doi: 10.1186/s12885-024-12120-0 (PMC10953198; doi:10.1186/s12885-024-12120-0)

Figure 1B gp130


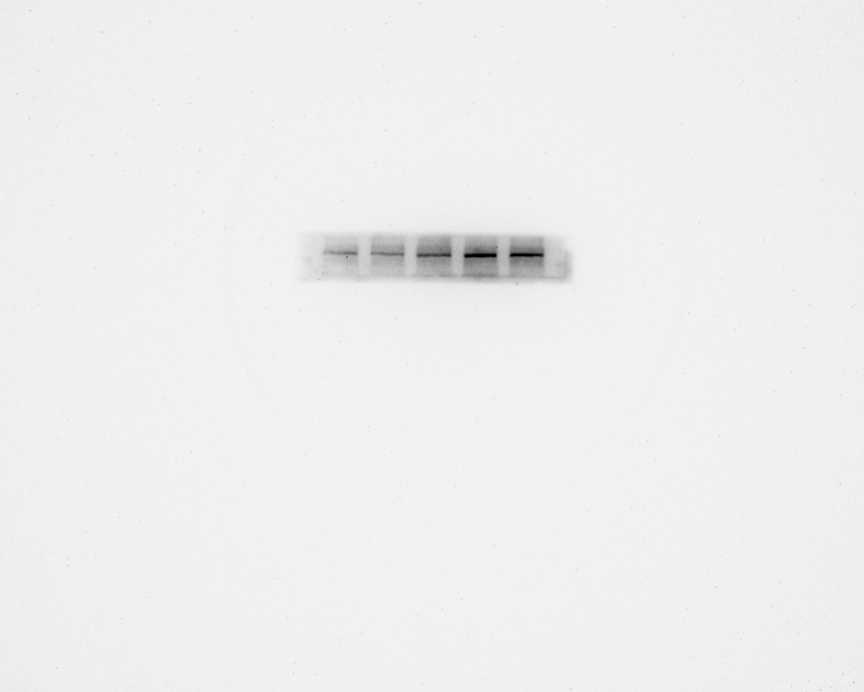


Figure 1B p-STAT3


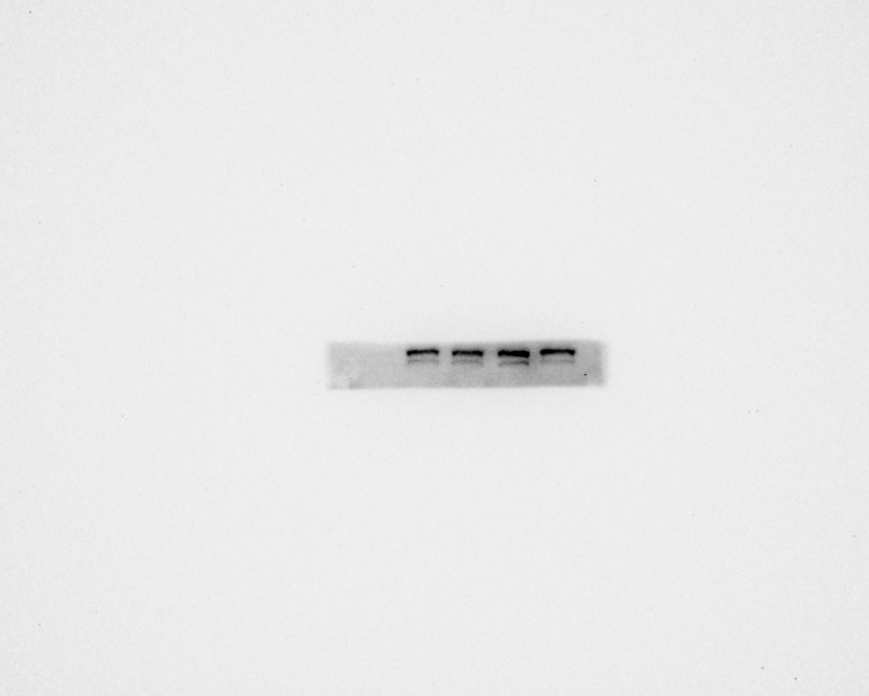


Figure 1B STAT3


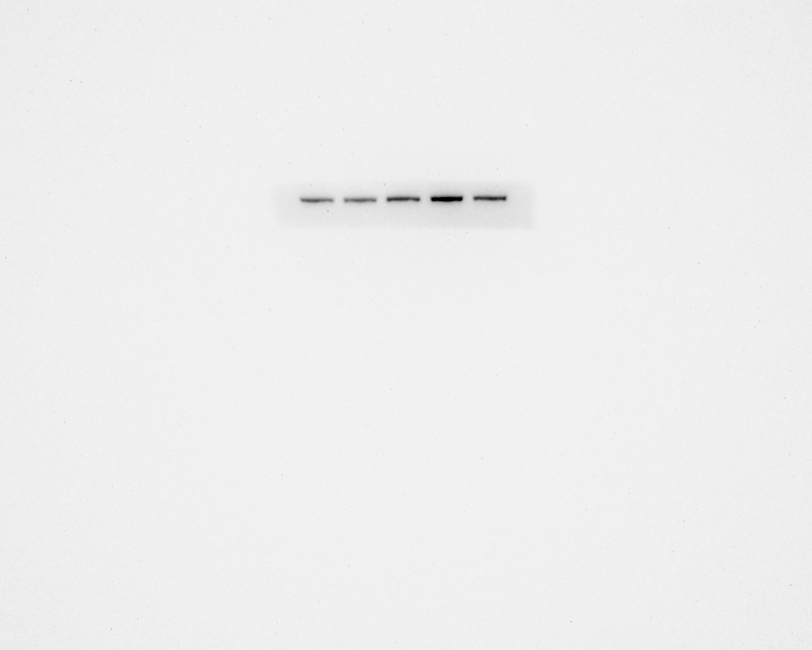


Figure 1B β-actin


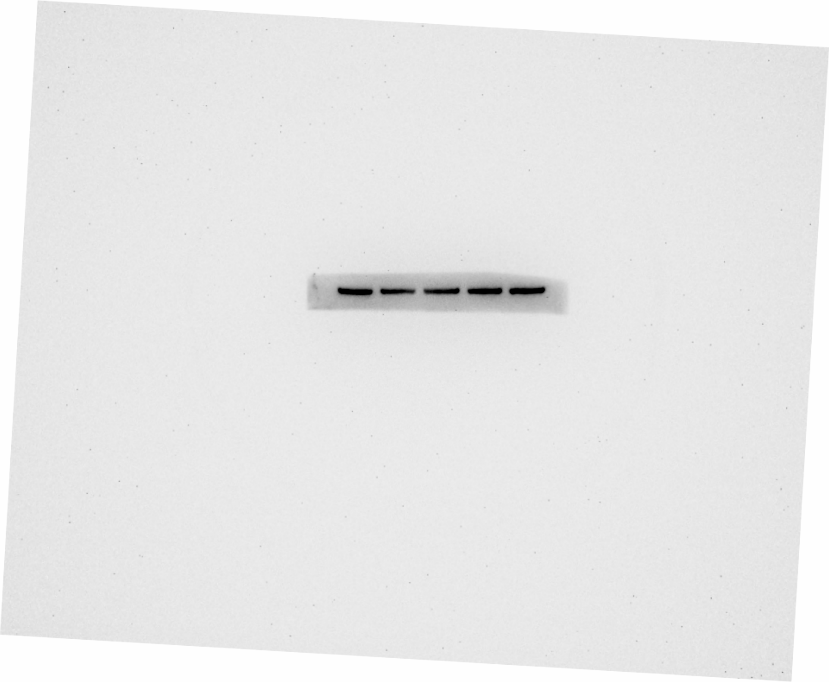


Figure 1C Bcl-xL


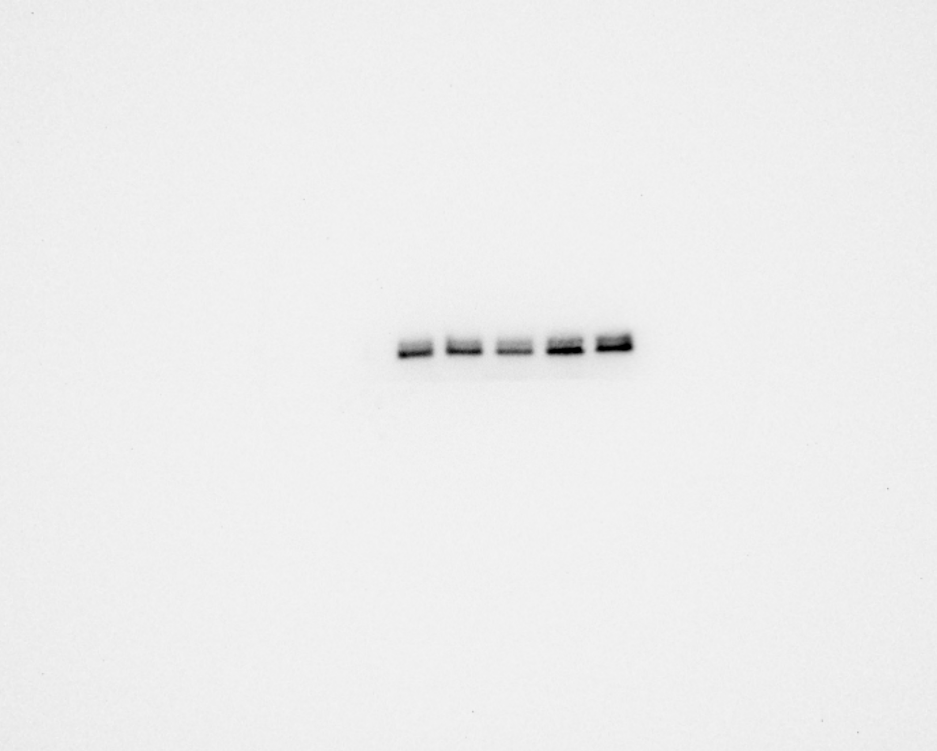


Figure 1C c-Myc


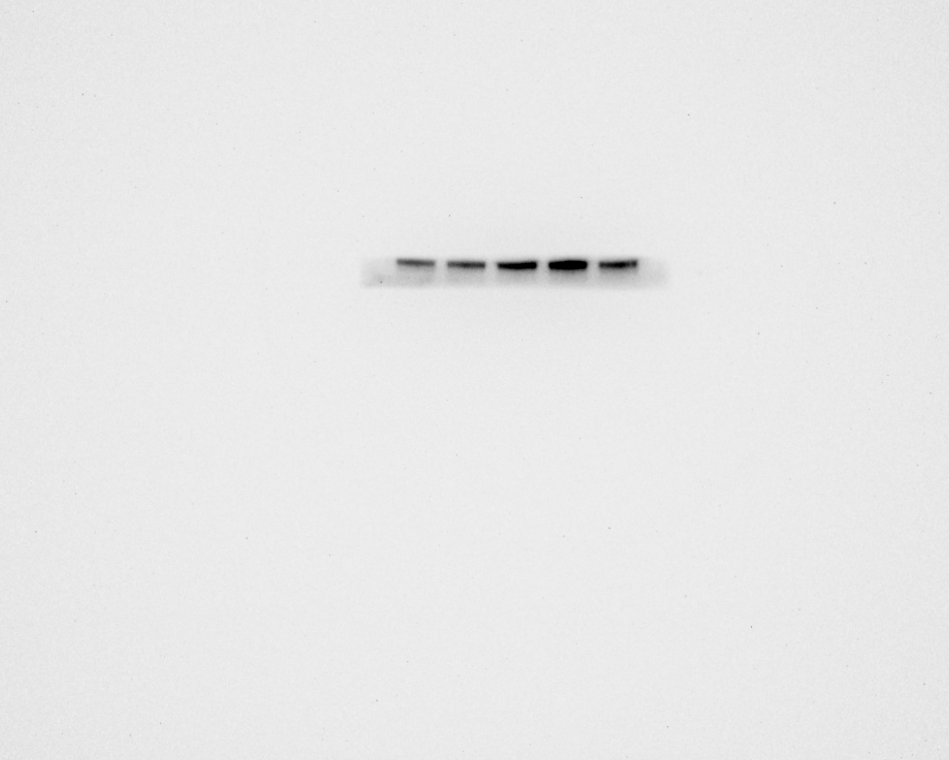


Figure 1C cyclin D1


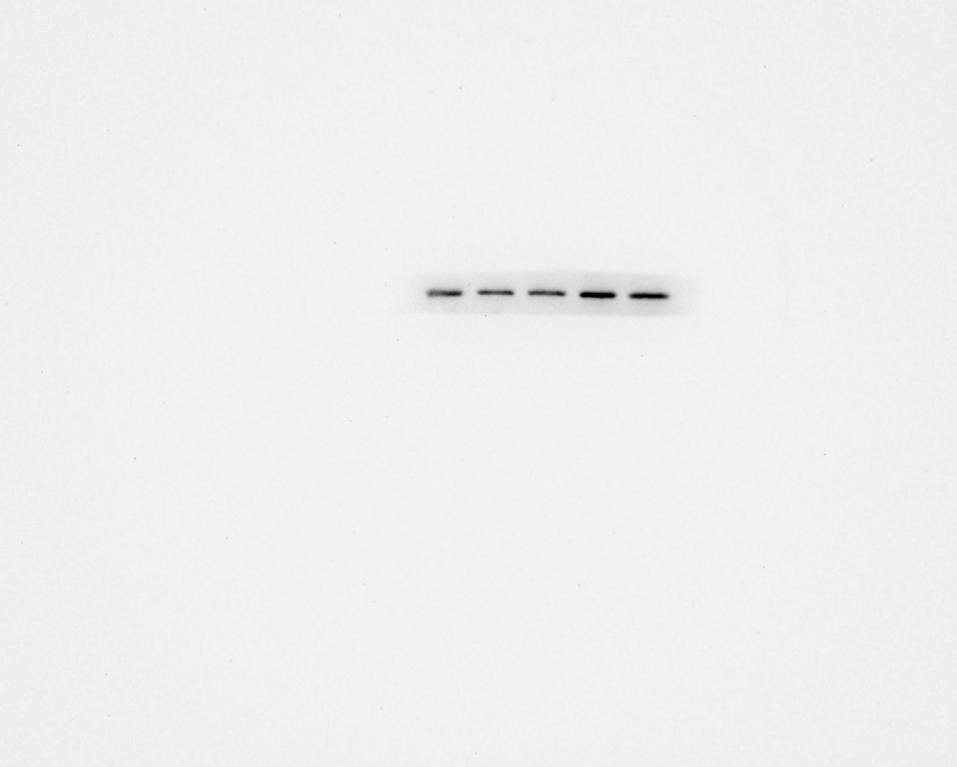


Figure 1C β-actin


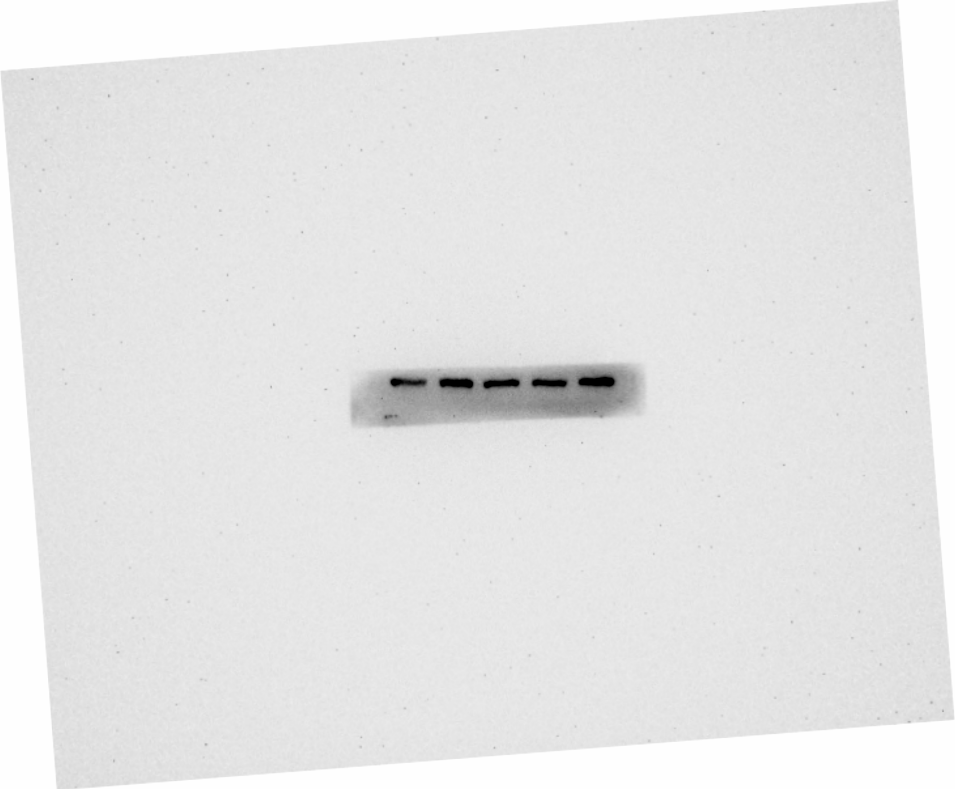


Figure 2B CT26 ART1


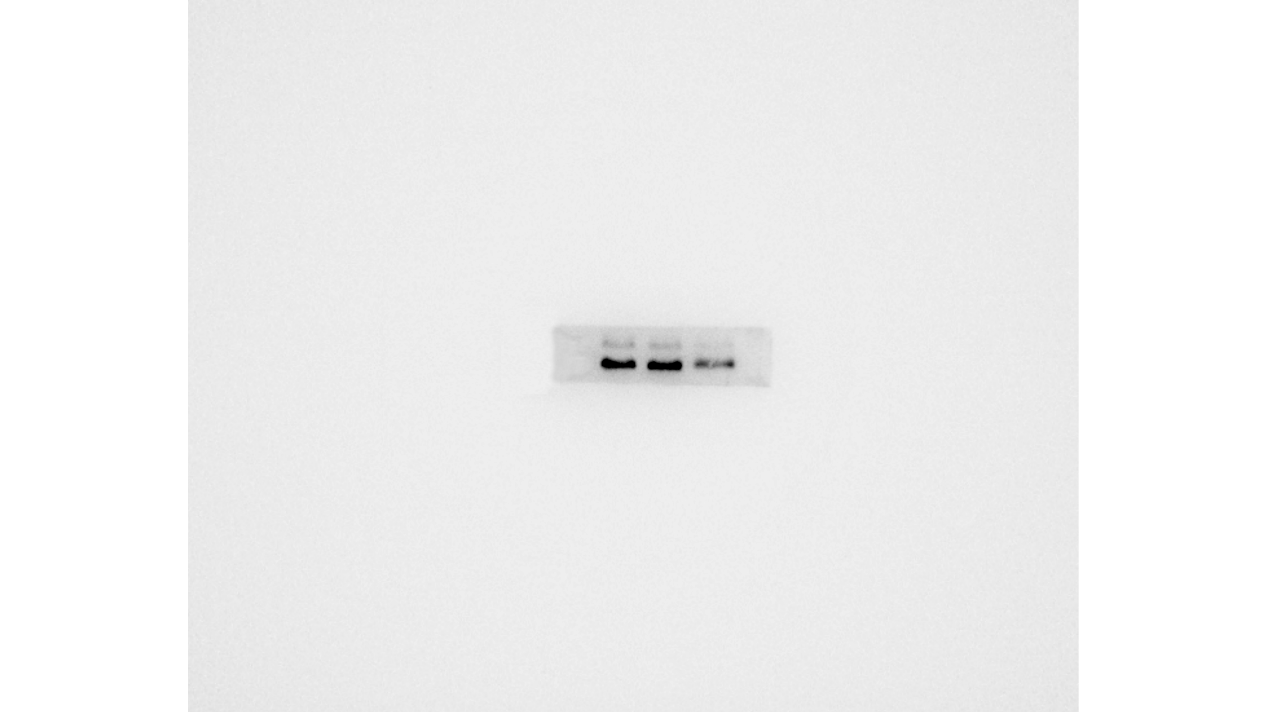


Figure 2B CT26 β-actin


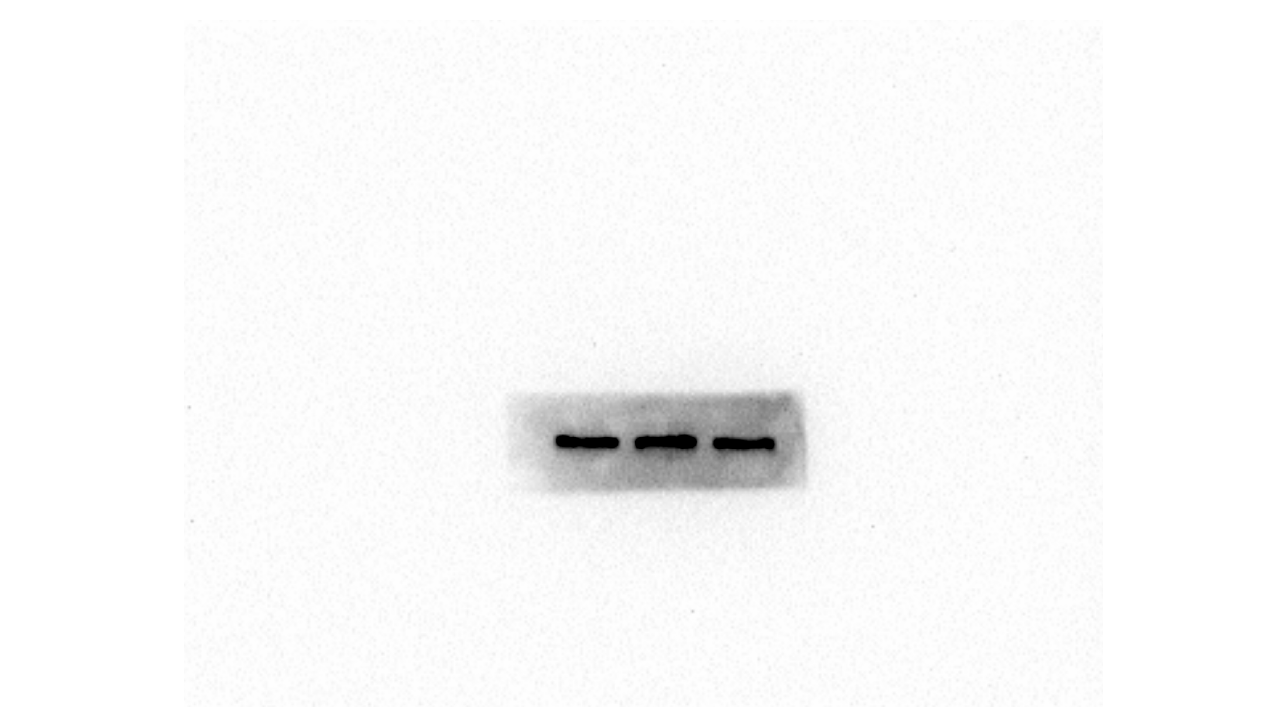


Figure 2B LoVo ART1


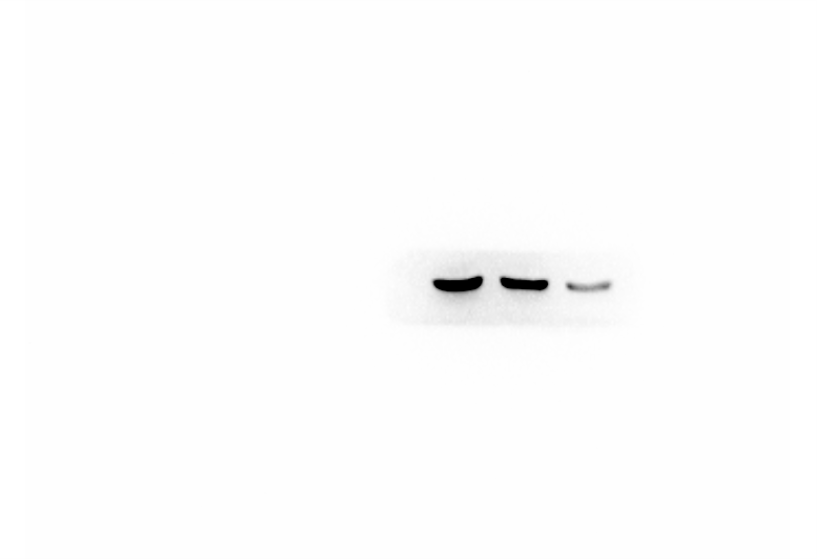


Figure 2B LoVo β-actin


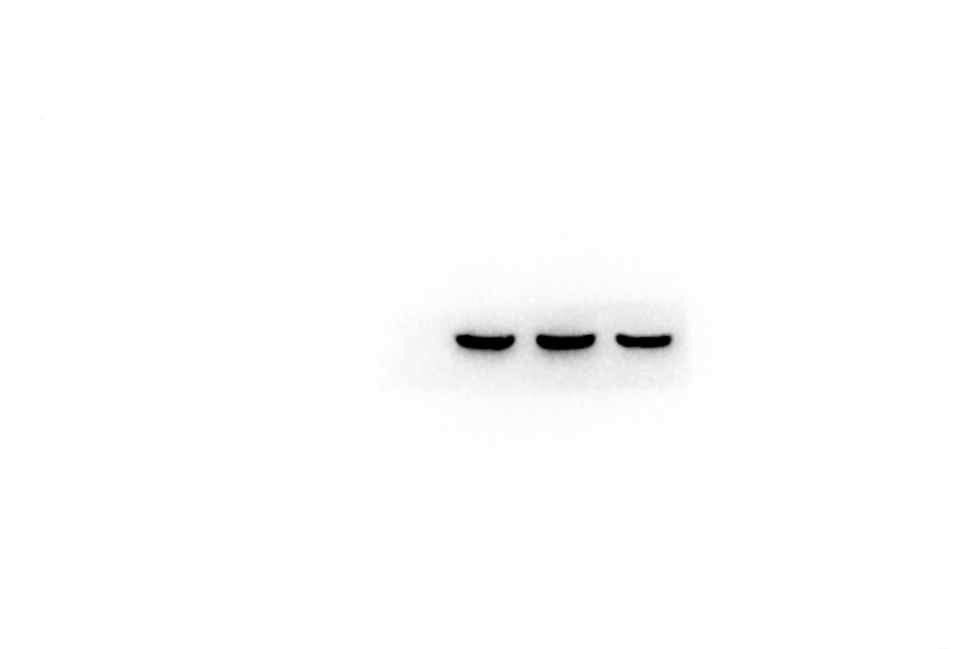


Figure 2D Bcl-xL


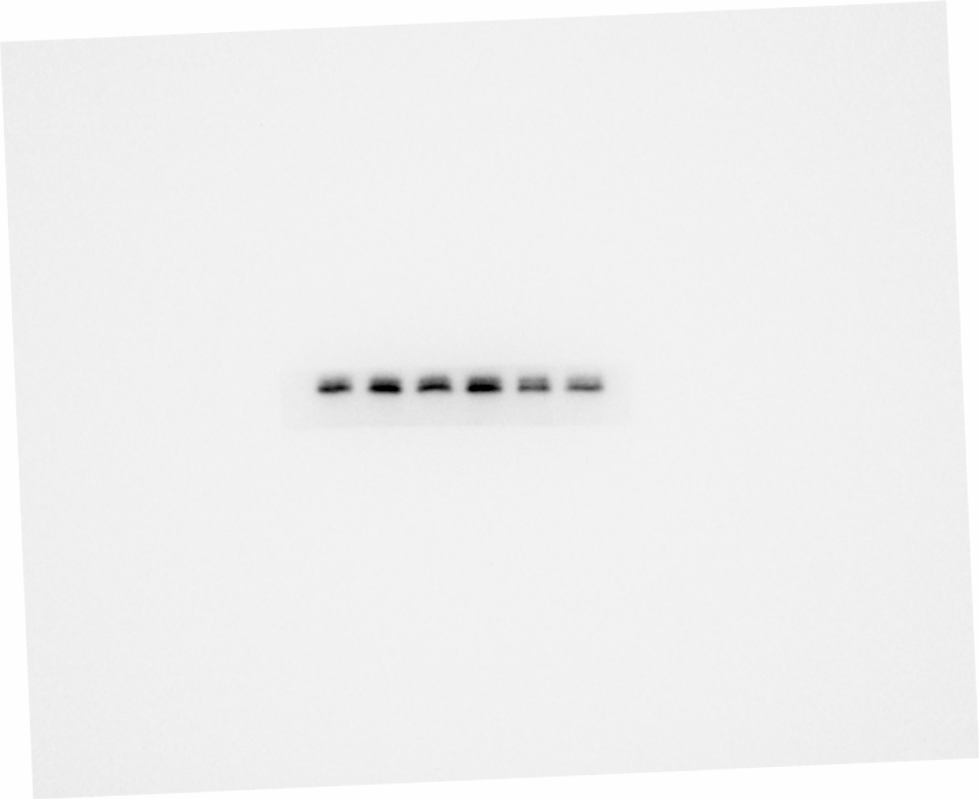


Figure 2D c-Myc


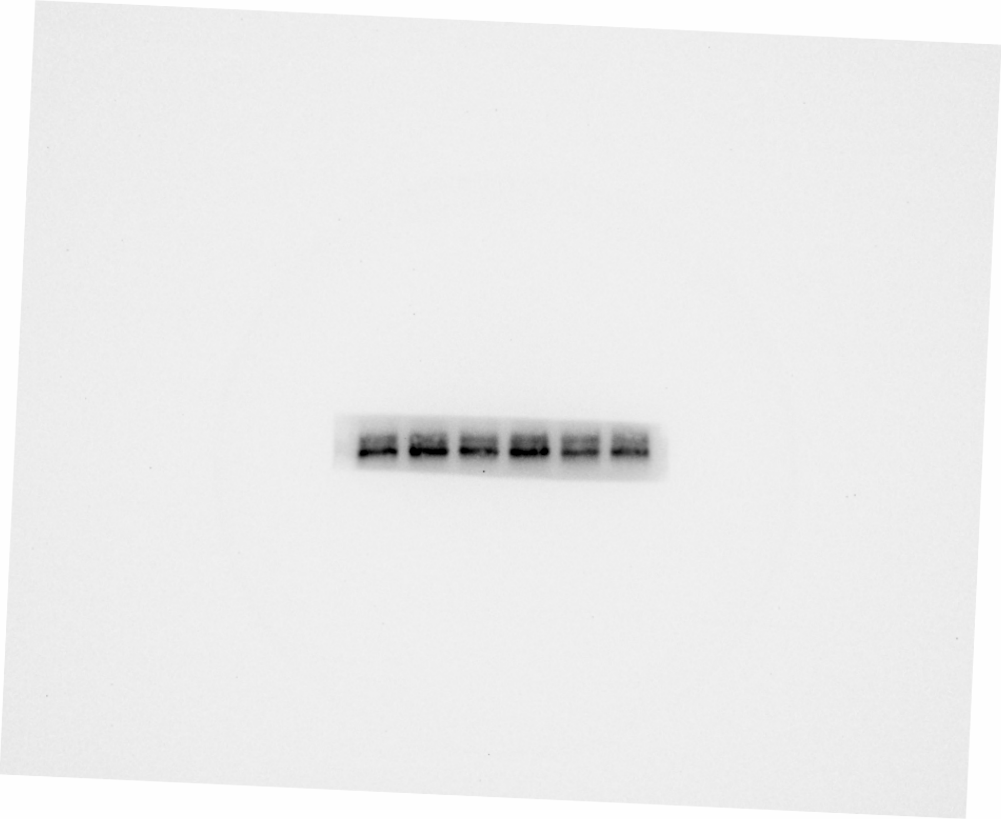


Figure 2D cyclin D1


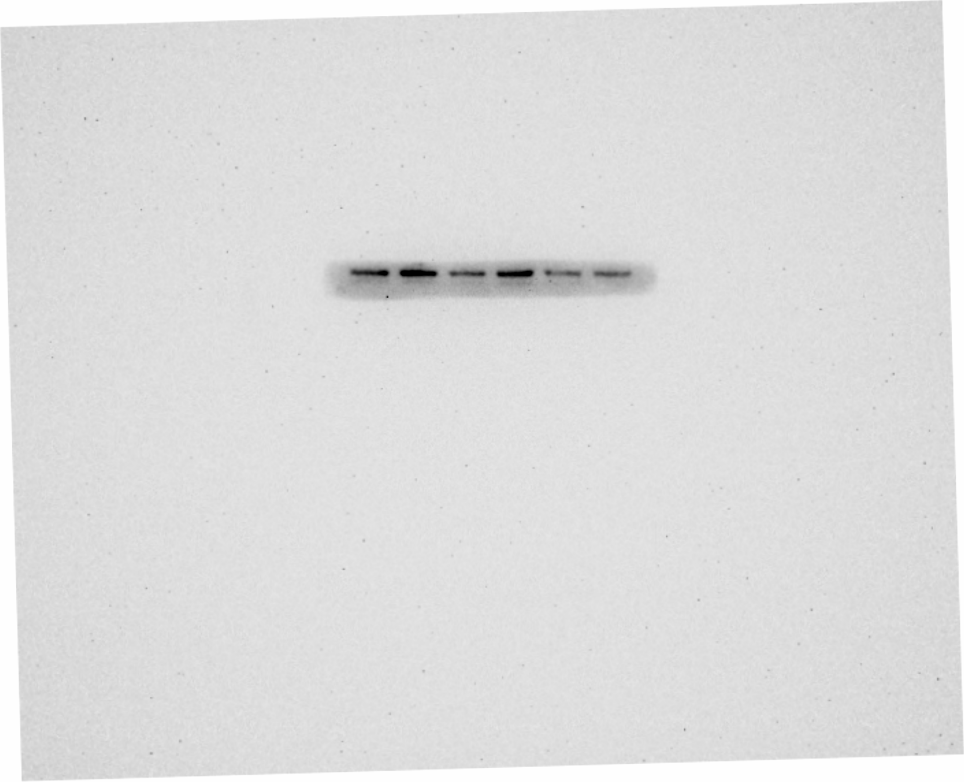


Figure 2D β-actin


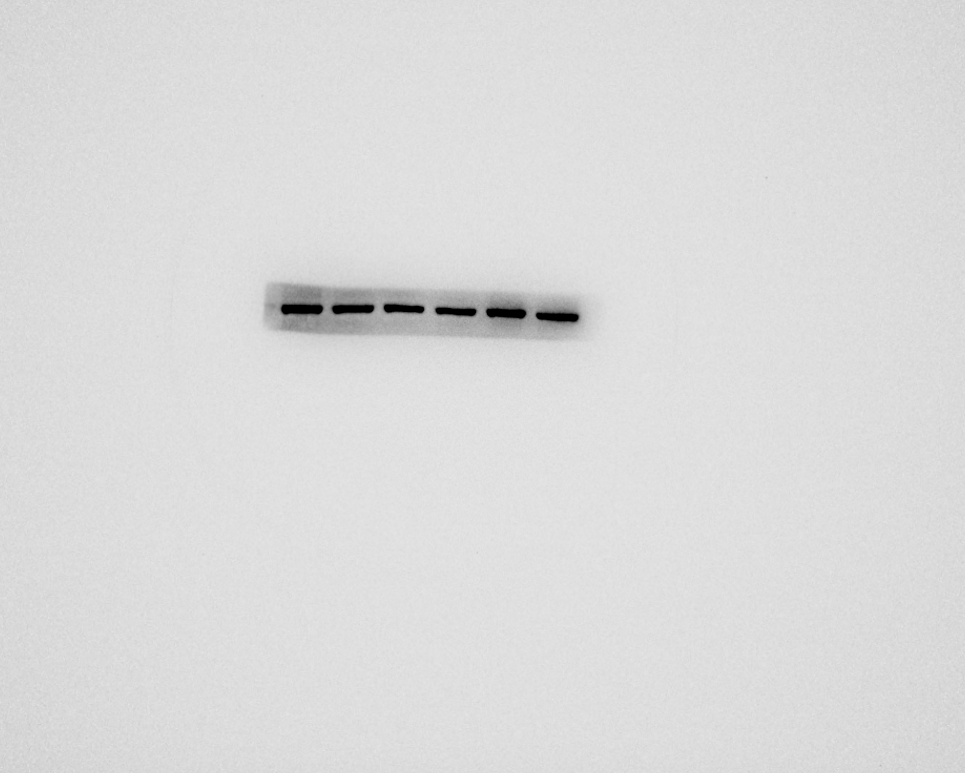


Figure 3A gp130


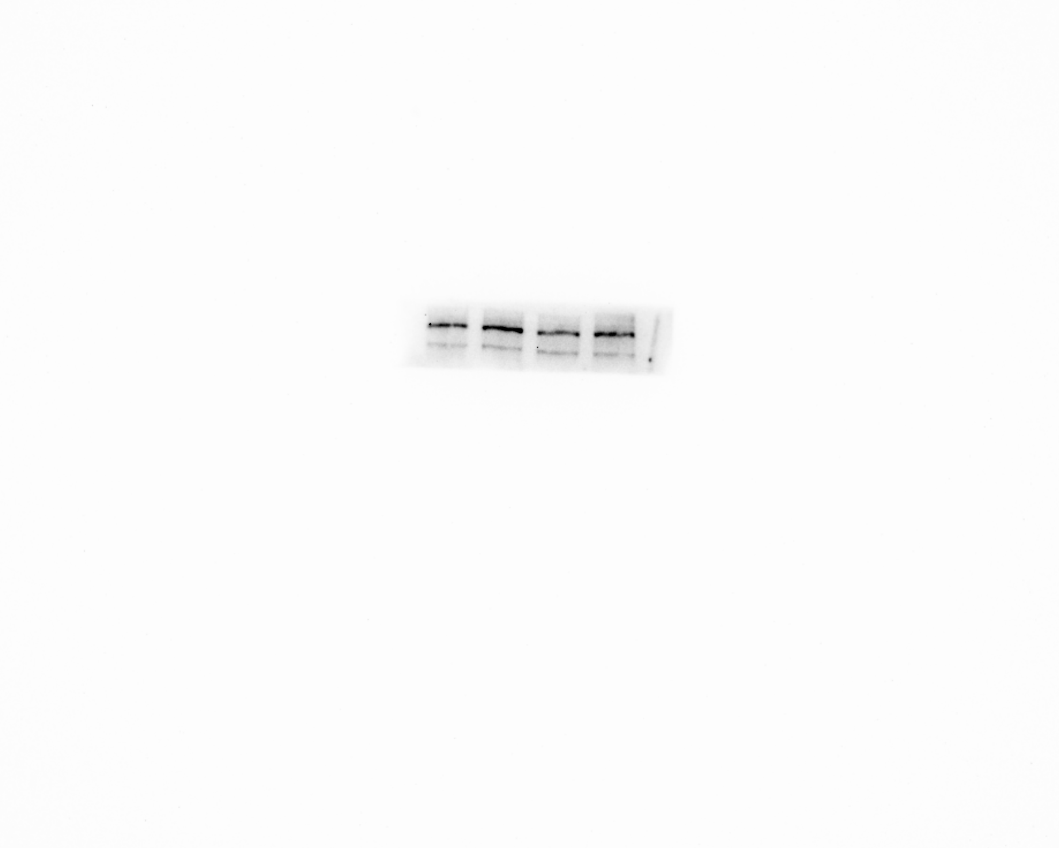


Figure 3A p-STAT3


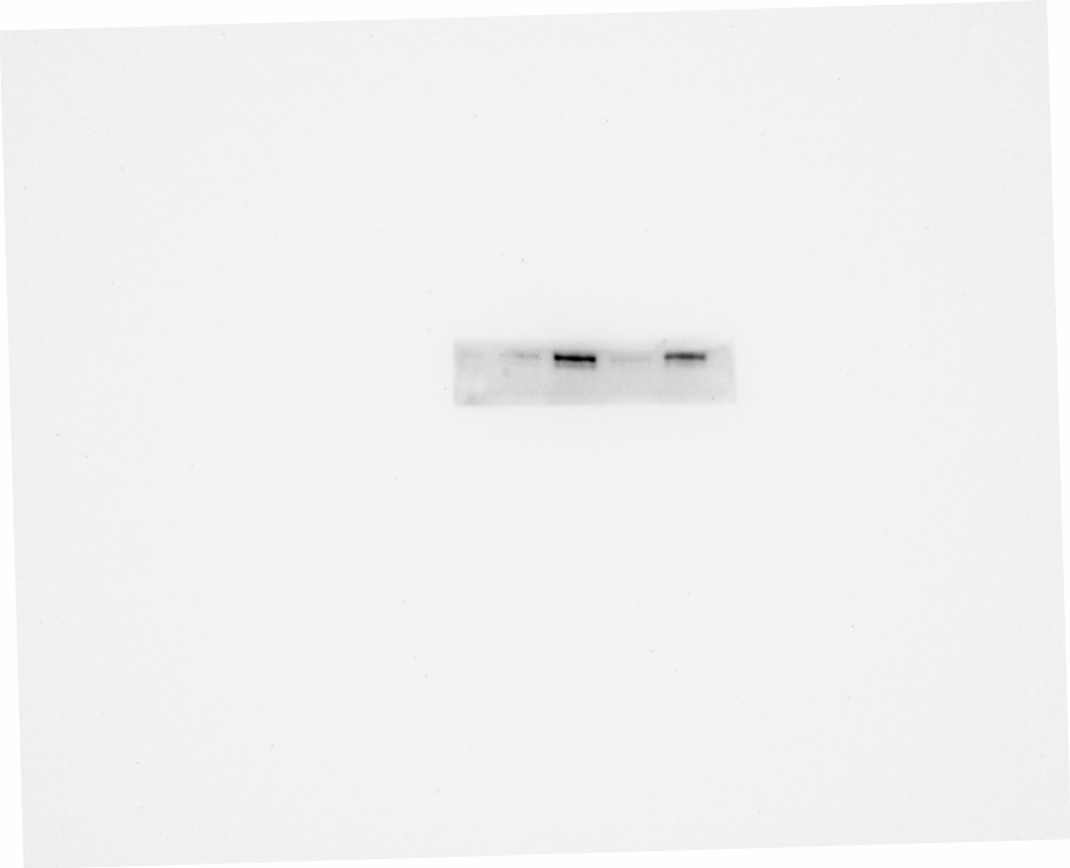


Figure 3A STAT3


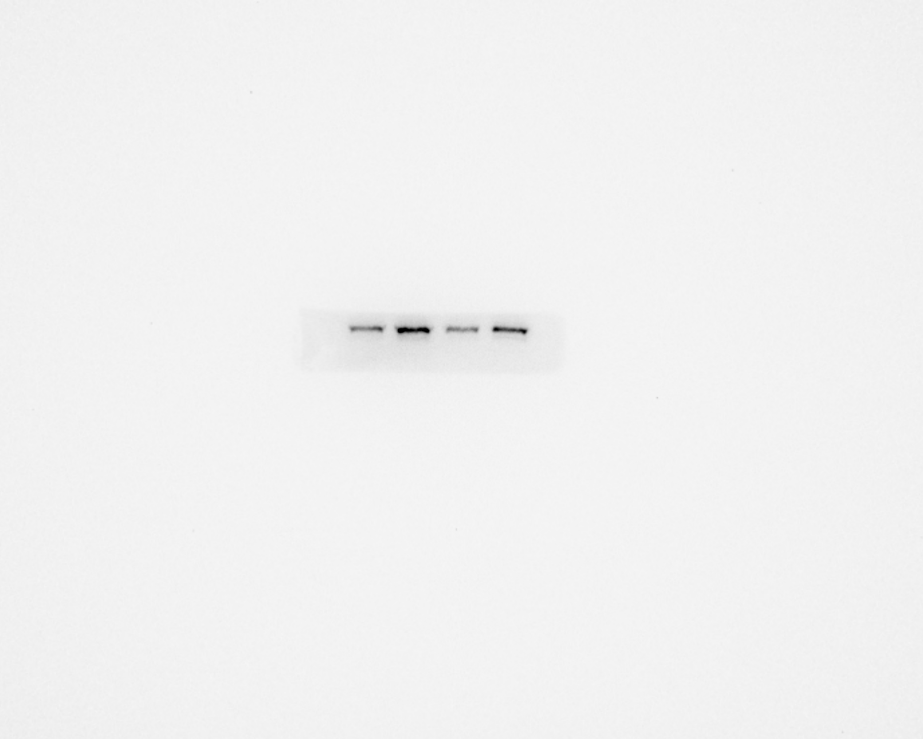


Figure 3A β-actin


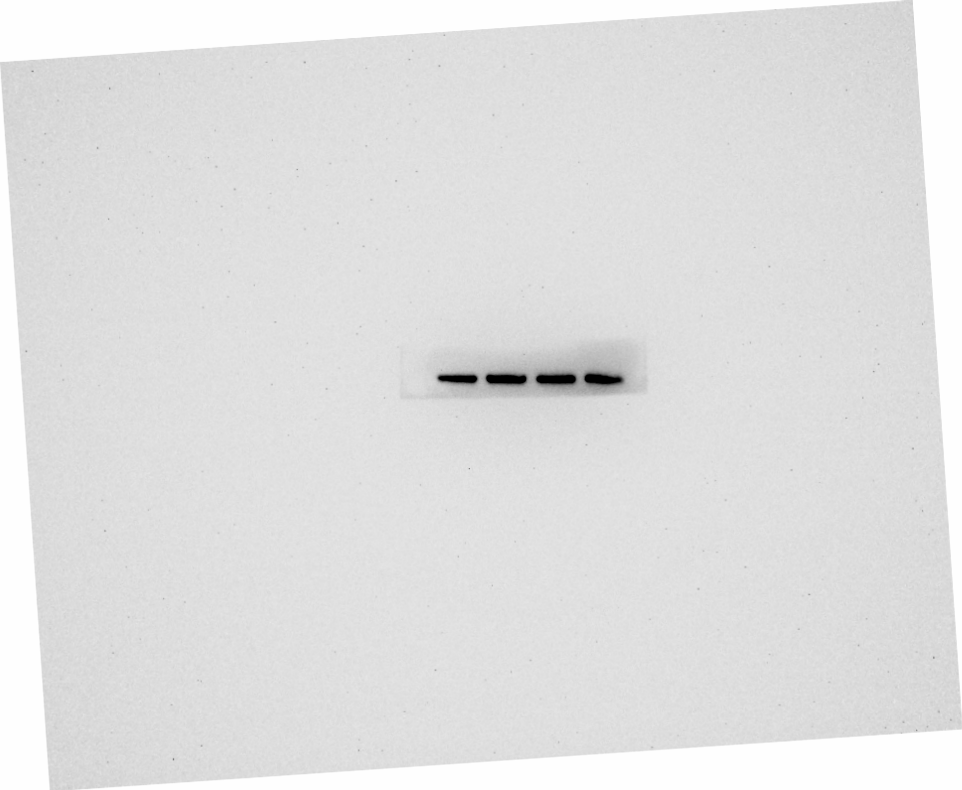


Figure 3B gp130


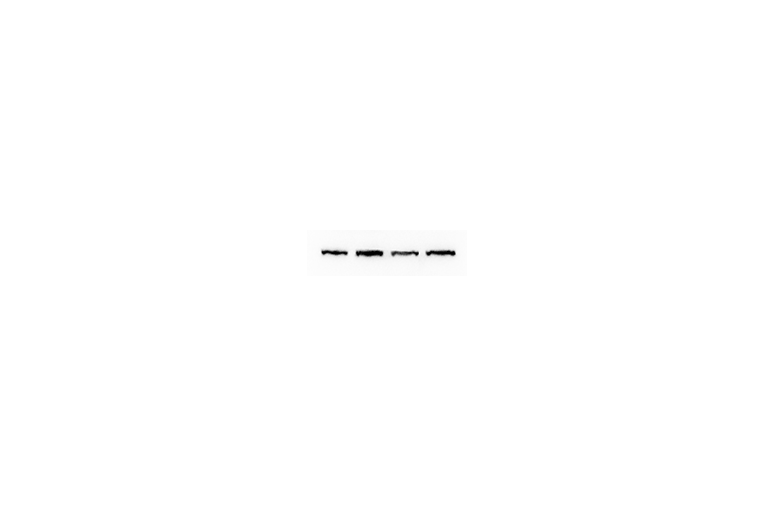


Figure 3B p-STAT3


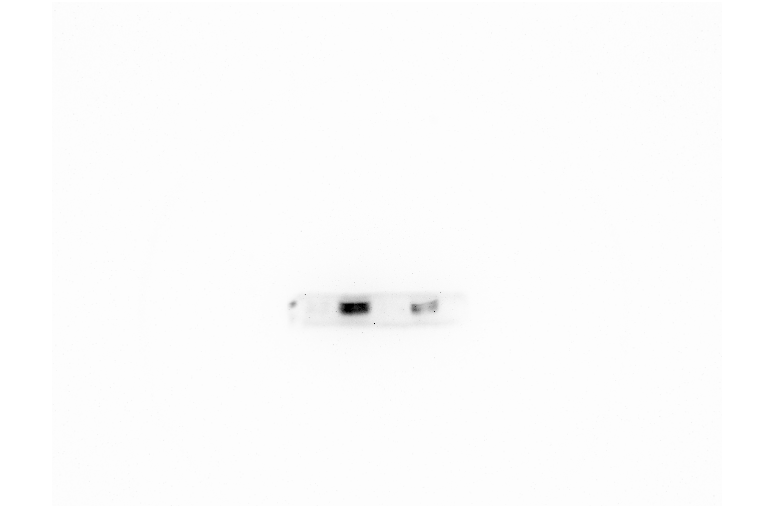


Figure 3B STAT3


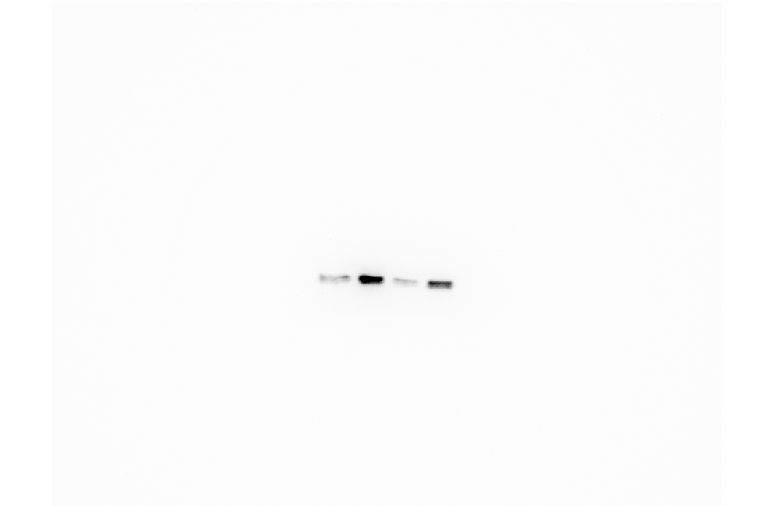


Figure 3B β-actin


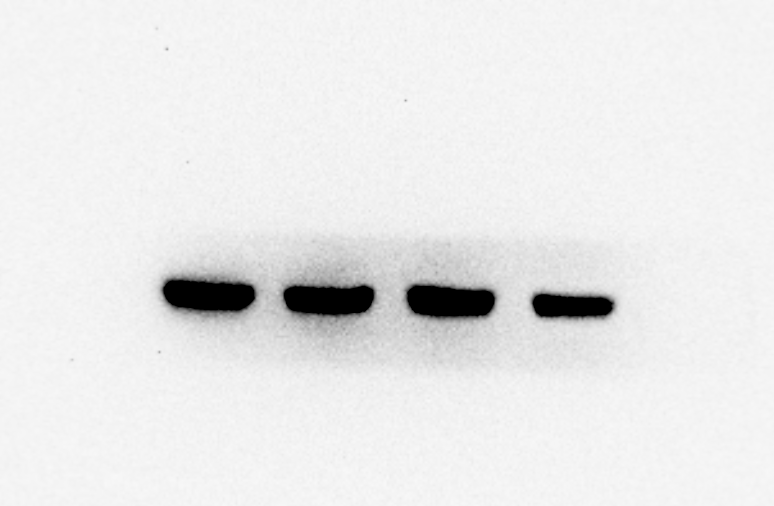


Figure 4A gp130


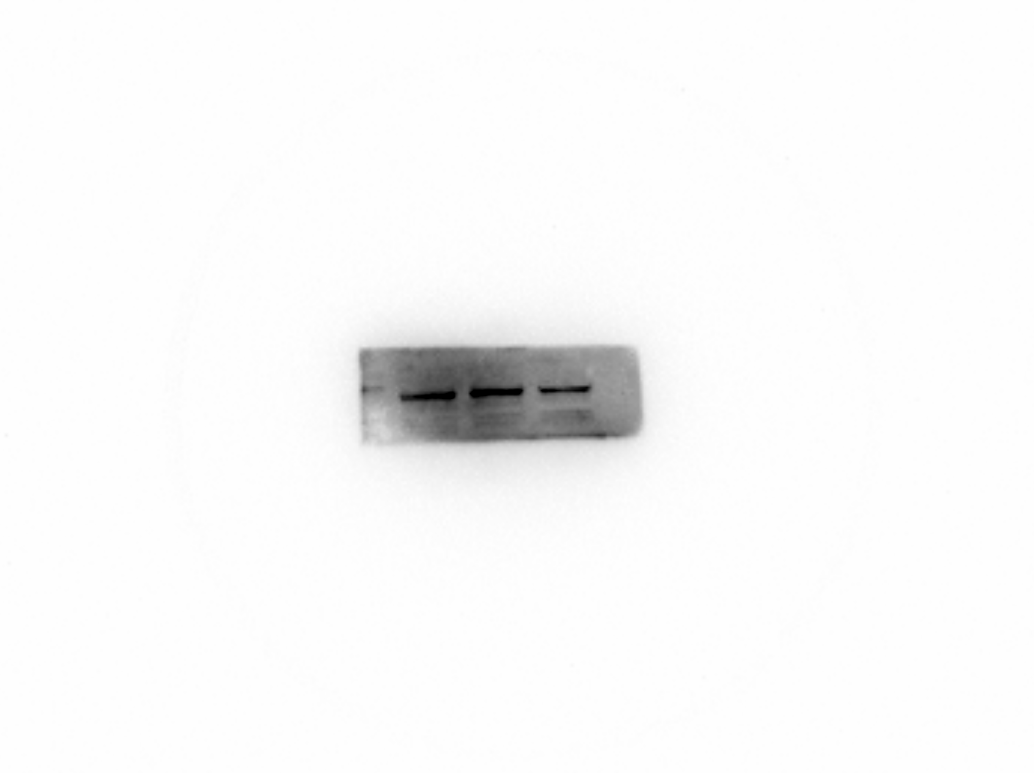


Figure 4A p-STAT3


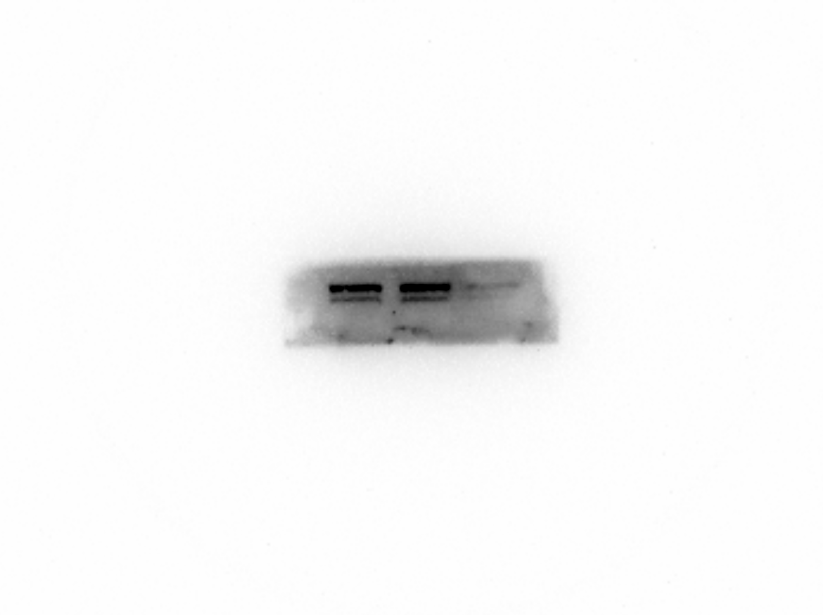


Figure 4A STAT3


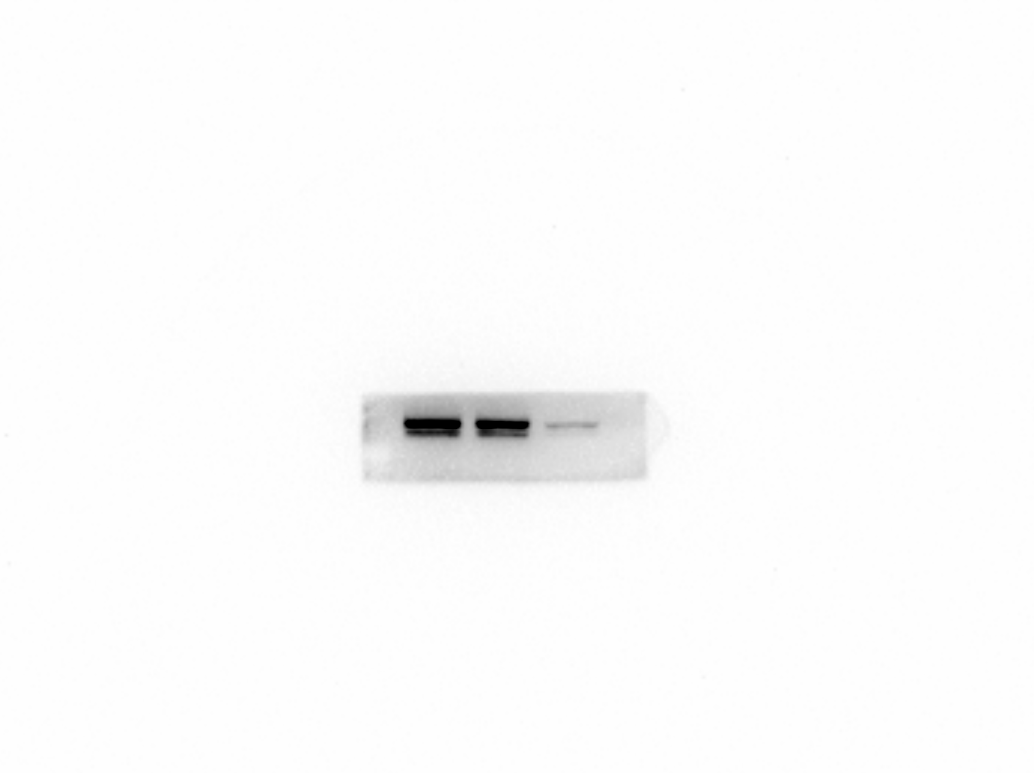


Figure 4A β-actin


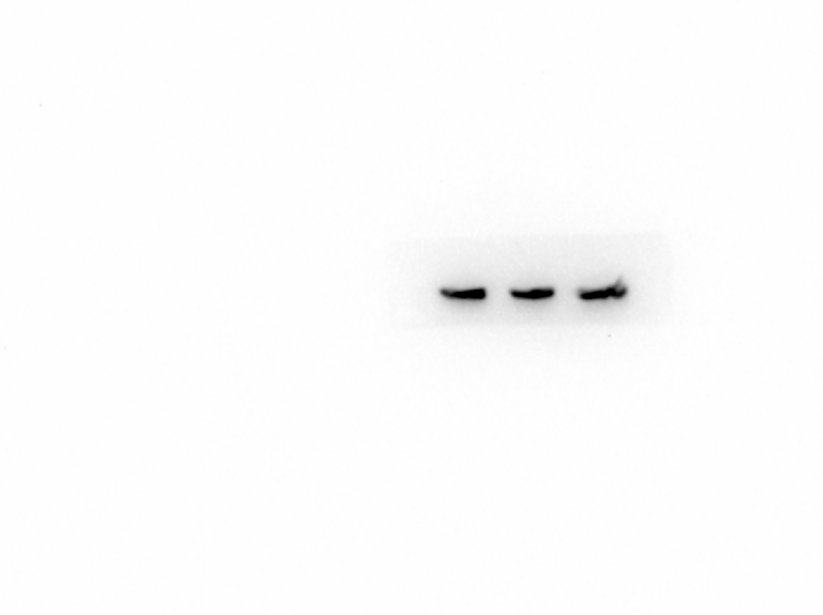


Figure 4B gp130


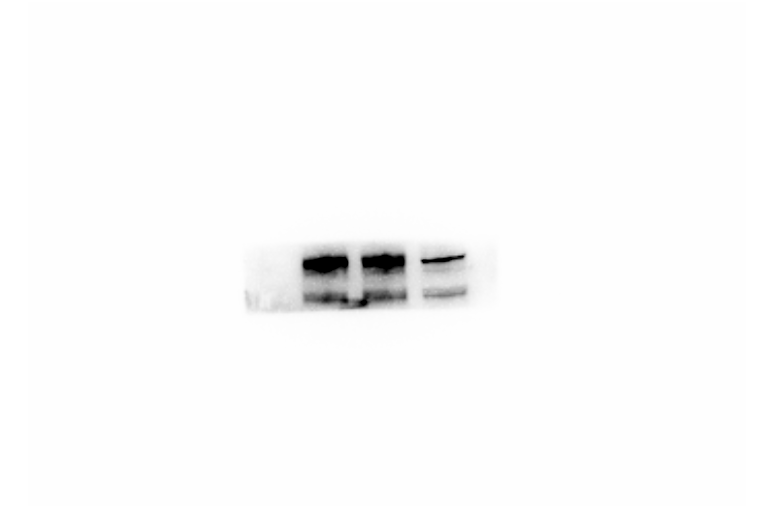


Figure 4B p-STAT3


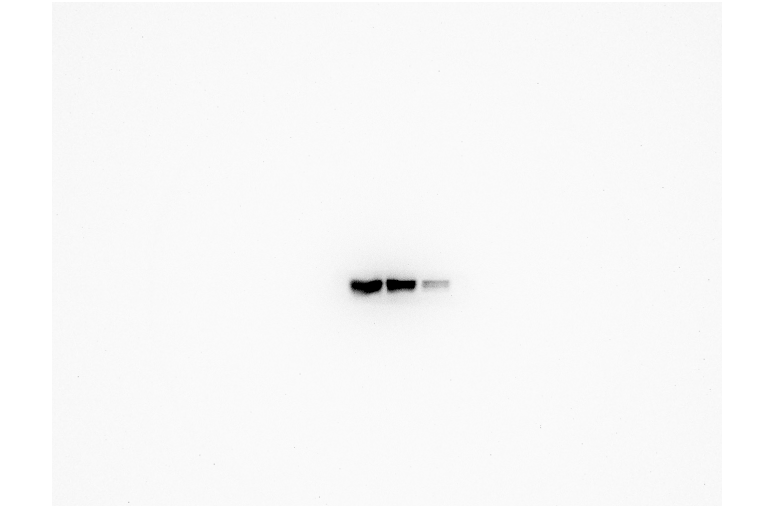


Figure 4B STAT3


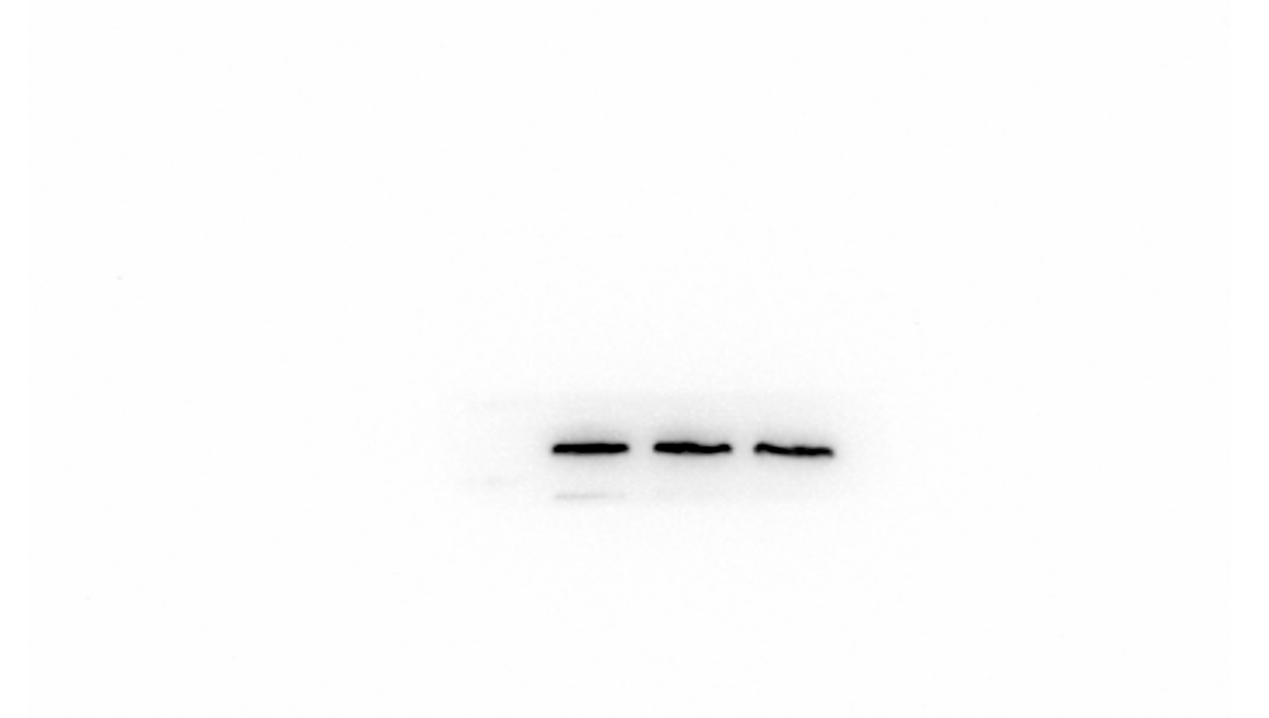


Figure 4B β-actin


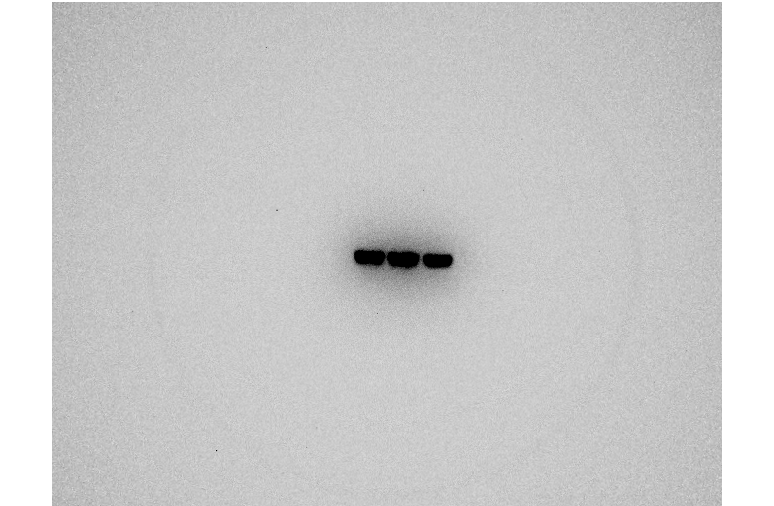


Figure 7D Bcl-xL


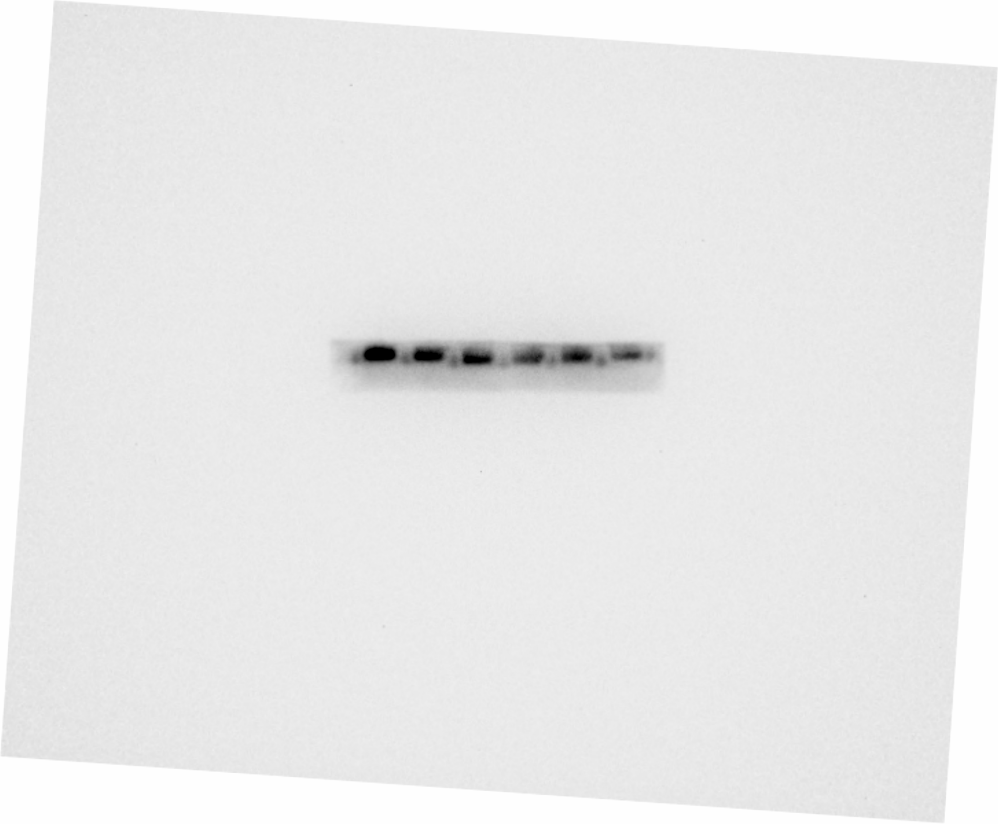


Figure 7D c-Myc


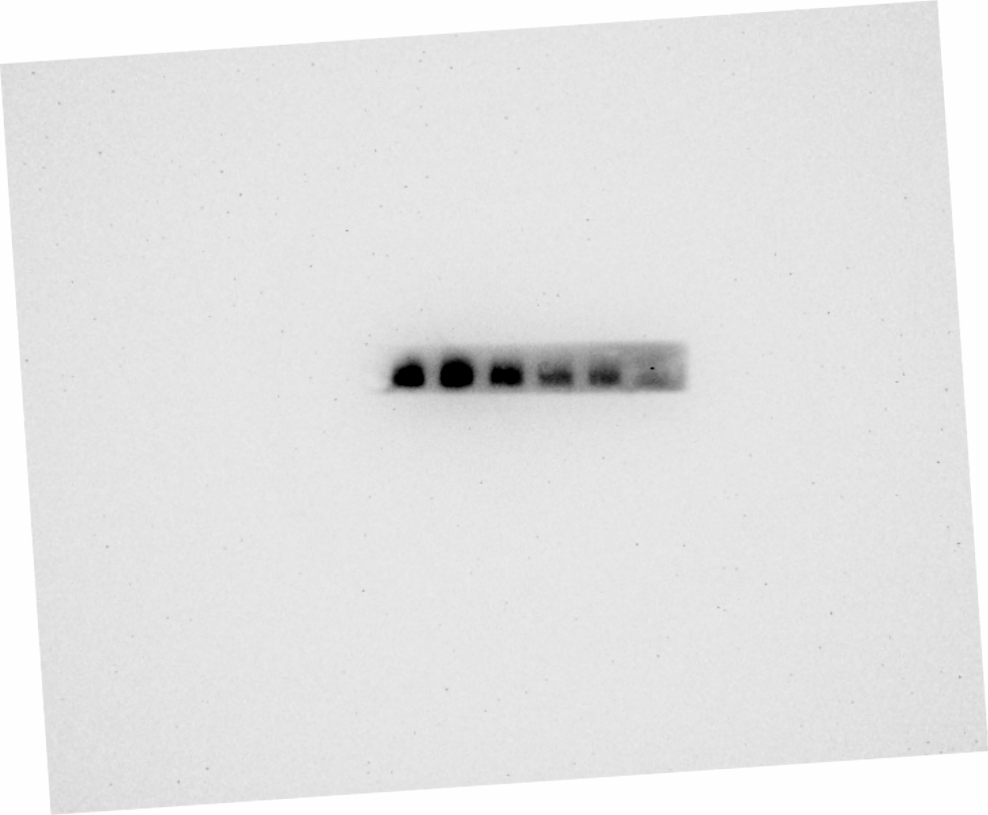


Figure 7D cyclin D1


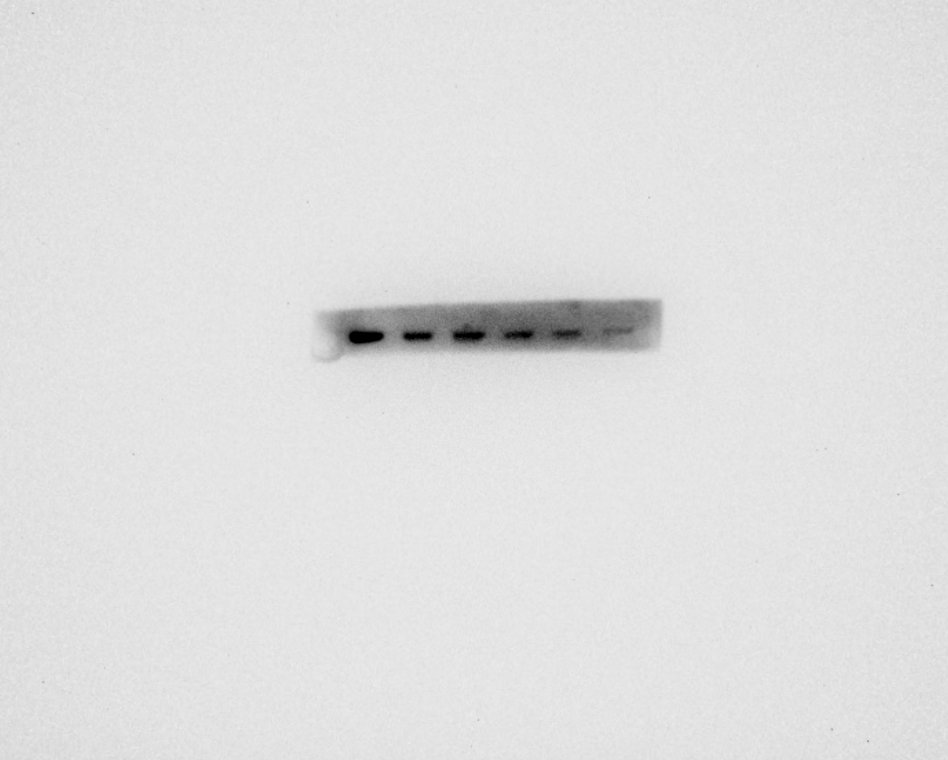


Figure 7D gp130


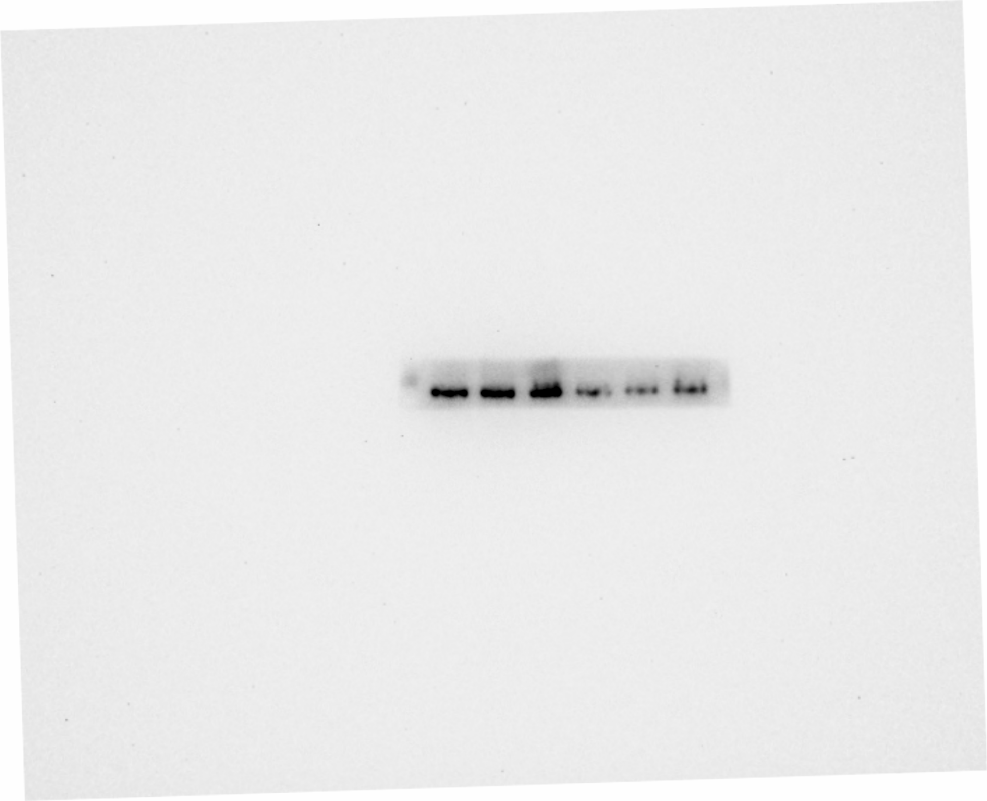


Figure 7D p-STAT3


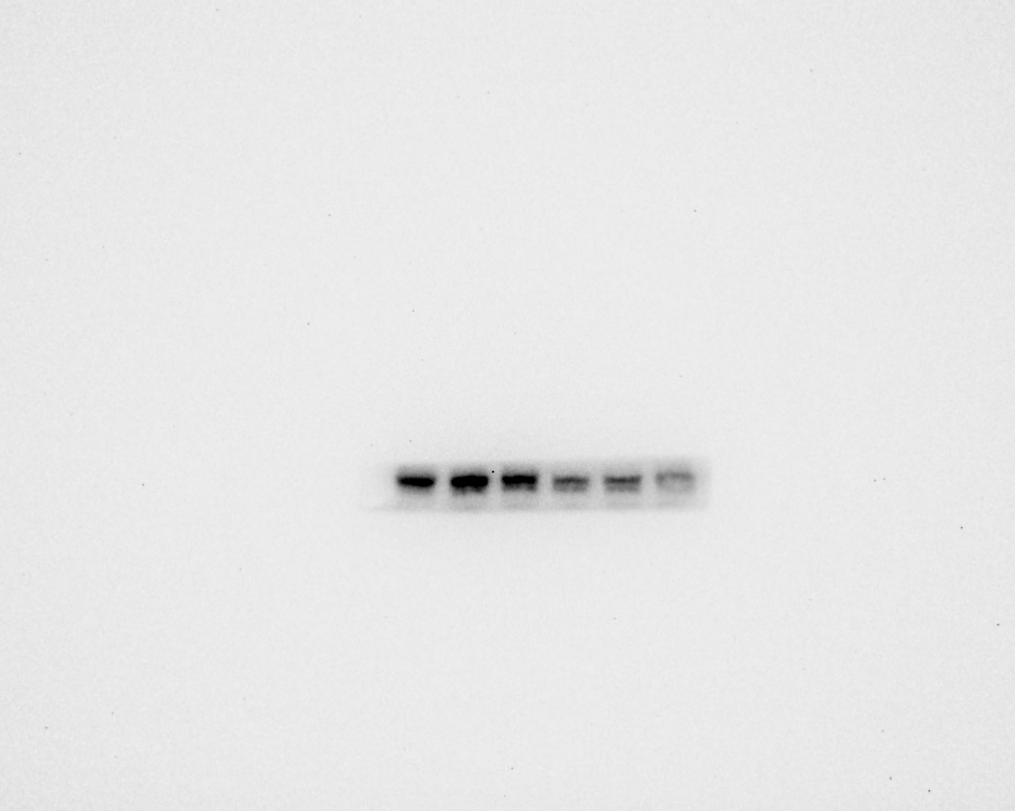


Figure 7D STAT3


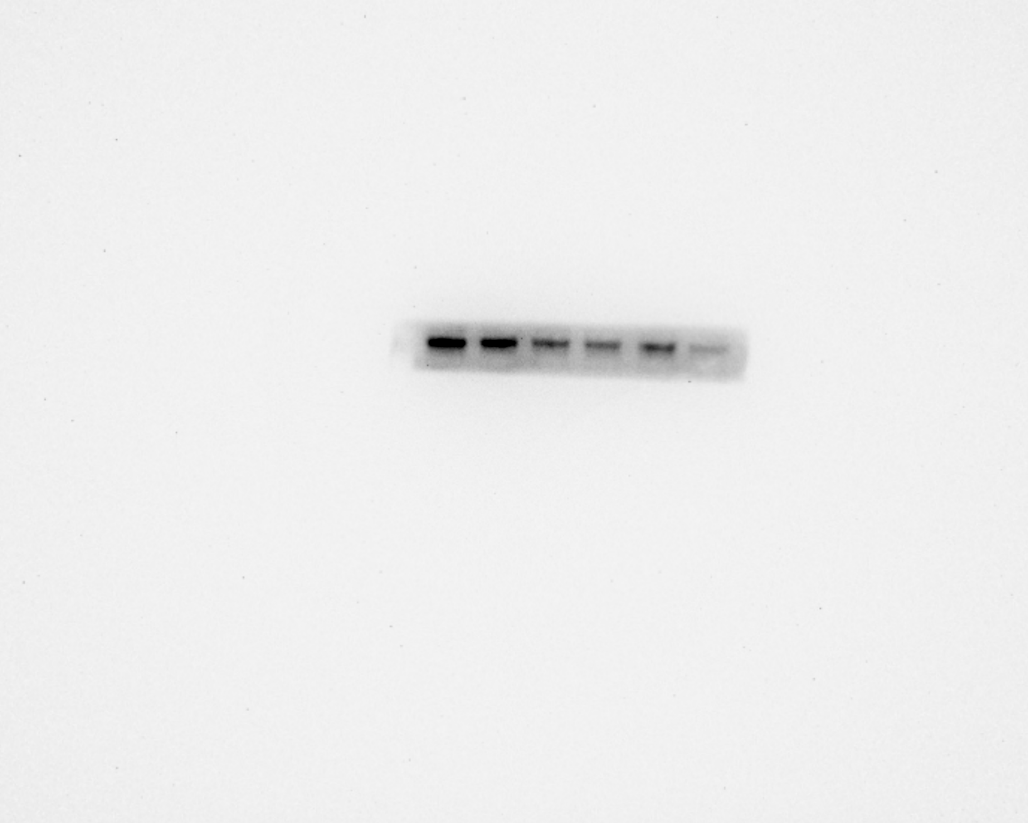


Figure 7D β-actin


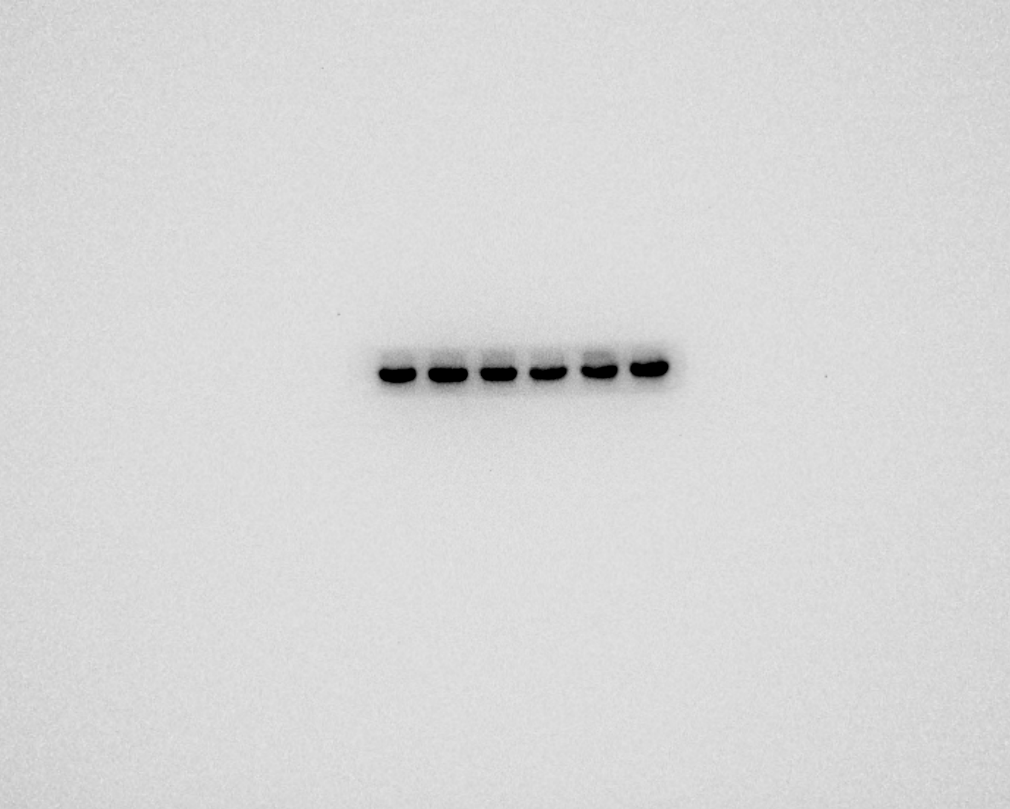

Supplement: Supplementary file 1 — Supplementary Material 1 [file 12885_2024_12120_MOESM1_ESM.docx]
